# Stability of Single Gold Atoms on Defective and Doped Diamond Surfaces

**DOI:** 10.1021/acs.jpcc.3c03900

**Published:** 2023-08-07

**Authors:** Shayantan Chaudhuri, Andrew J. Logsdail, Reinhard J. Maurer

**Affiliations:** † Department of Chemistry, University of Warwick, Coventry CV4 7AL, United Kingdom; ‡ Centre for Doctoral Training in Diamond Science and Technology, University of Warwick, Coventry CV4 7AL, United Kingdom; § Cardiff Catalysis Institute, School of Chemistry, Cardiff University, Cardiff CF10 3AT, United Kingdom; ∥ Department of Physics, University of Warwick, Coventry CV4 7AL, United Kingdom

## Abstract

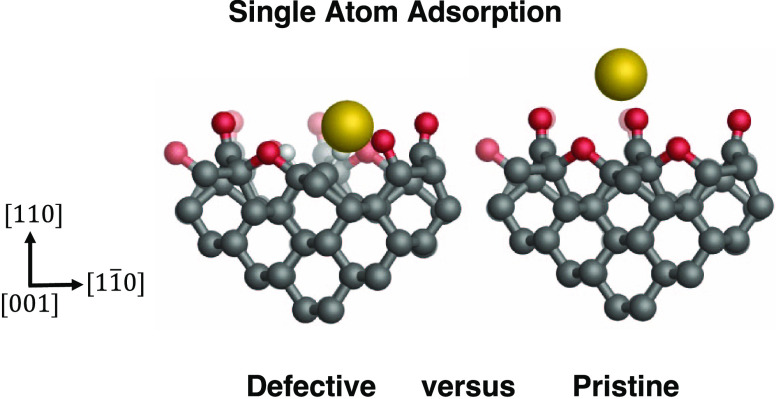

Polycrystalline boron-doped diamond (BDD) is widely used as a working electrode material in electrochemistry, and its properties, such as its stability, make it an appealing support material for nanostructures in electrocatalytic applications. Recent experiments have shown that electrodeposition can lead to the creation of stable small nanoclusters and even single gold adatoms on the BDD surfaces. We investigate the adsorption energy and kinetic stability of single gold atoms adsorbed onto an atomistic model of BDD surfaces by using density functional theory. The surface model is constructed using hybrid quantum mechanics/molecular mechanics embedding techniques and is based on an oxygen-terminated diamond (110) surface. We use the hybrid quantum mechanics/molecular mechanics method to assess the ability of different density functional approximations to predict the adsorption structure, energy, and barrier for diffusion on pristine and defective surfaces. We find that surface defects (vacancies and surface dopants) strongly anchor adatoms on vacancy sites. We further investigated the thermal stability of gold adatoms, which reveals high barriers associated with lateral diffusion away from the vacancy site. The result provides an explanation for the high stability of experimentally imaged single gold adatoms on BDD and a starting point to investigate the early stages of nucleation during metal surface deposition.

## Introduction

The design of novel materials for electrocatalytic applications is driven by the need to achieve high activity and selectivity for catalytic reactions that are crucial to improving sustainability in industrial processes. Noble-metal nanomaterials that are based on gold and its alloys are emerging as efficient heterogeneous electrocatalysts due to their stability, versatility, and lower cost compared to platinum- and rhodium-based electrocatalysts. Furthermore, gold nanoclusters are known to adopt unique electronic and geometric structures.^1,2^ The electrocatalytic activity of monometallic,^3−9^ bimetallic,^10−20^ and multimetallic^21−23^ gold-based nanostructures has been well established in the literature. Metal nanostructures are typically created by deposition on supporting semiconductors and oxide thin films or nanoparticles. Metal deposition naturally starts with the adsorption of single metal atoms,^24−27^ which are also, thus, the starting point for the growth of larger nanostructures. Single metal atoms have been shown to have unique magnetic properties^28^ and excellent (electro)catalytic applications;^29−32^ indeed, single-atom catalysts can outperform larger nanostructures due to their optimal atom utilization.^33−35^ Supported single gold atoms in particular have been shown to be very efficient electrocatalysts for a variety of key chemical processes, including nitrogen reduction^29−31^ and oxygen reduction and evolution.^32^ The potential impact of these single-gold-atom catalysts makes it essential to investigate the variety of possible stabilization mechanisms that can promote the successful deposition of single gold atoms onto surfaces. Furthermore, as much still remains unclear about the early stages of metal deposition and the role of the atomic-scale structure on the surface,^36^ investigating the adsorption of single metal atoms can provide some key insights into the initial stages of nanocluster formation and nucleation.

The structure and reactivity of nanostructures depend on the nature and morphology of the support, which affects the interaction between the adsorbate and the support surface and also influences the structural and electronic properties exhibited by the nanostructure.^37,38^ The adsorption of gold atoms has been investigated on a variety of supports such as magnesium oxide,^39−41^ cerium(IV) oxide,^41−47^ and graphene/graphite.^48−54^ Boron-doped diamond (BDD), in particular, is an attractive support material for electrocatalytic applications due to its high stability and electrical conductivity.^55−58^ The controlled formation of gold nanostructures on BDD has recently been reported,^36,59,60^ which has enabled interesting electroanalytical^61−67^ and electrocatalytic applications.^4^ Hussein et al. reported the electrochemical deposition of small nascent nanoclusters and single gold atoms on BDD surfaces; using identical-location scanning transmission electron microscopy (STEM), single gold atoms were shown to be stable atop polished polycrystalline BDD surfaces.^36^ The study reported that single atoms were stable in their original adsorption sites despite considerable momentum transfer from repeated STEM measurements in the same area. The same study found that the diffusion barriers for single gold atoms on idealized oxygen-terminated BDD surfaces, composed of coexistent carbonyl and ether groups, are too low to be consistent with the high stability observed in the STEM experiments.^36^ The result suggests that the observed stability of single atoms is likely due to defects and dopants on the BDD surfaces that are not visible in the STEM images and that were not accounted for within the original electronic structure calculations.

First-principles methods such as density functional theory (DFT)^68,69^ can provide detailed insight into the structural and electronic properties of supported metal atoms,^37,70,71^ and how they are affected by the atomic-scale structure of the substrate surface. However, periodic surface slab models often exhibit poor computational scaling behavior^72^ that limits the application of more accurate higher-rung density functional approximations (DFAs)^73^ when studying large, periodic models.^74^ Due to the exhaustive computational requirements, the choice of DFA is often limited in large-scale studies to generalized gradient approximations (GGAs) or meta-GGAs (MGGAs) when calculating the Kohn–Sham ground-state energy.^70,71^ These DFAs typically estimate either the adsorption energy or the reaction barriers correctly, but rarely both.^70,71^ GGAs also often lack inclusion of long-range dispersion interactions, which are crucial for an accurate description of hybrid organic–inorganic interfaces.^70,71^ Long-range dispersion correction methods, such as the Grimme series of methods^75^ or many-body dispersion (MBD) approaches,^76−78^ are well-established strategies to address this shortcoming.

The challenges associated with periodic representation of defects can be overcome by creating truncated cluster models. However, this removes the long-range properties of any bulk material and such calculations can be plagued by spurious finite size effects.^71^ Embedded cluster calculations based on a hybrid quantum mechanics/molecular mechanics (QM/MM)^79,87^ methodology are a viable alternative to periodic slab calculations as they acknowledge that surface defect chemistry is intrinsically local. Embedded cluster models of extended surfaces allow for isolated point or charge defects to be modeled that break translational periodicity. Furthermore, QM/MM models are generally computationally cheaper, and higher-rung functionals are more straightforward to apply for the aperiodic case. Therefore, higher-rung DFAs, such as hybrid GGAs (HGGAs), become accessible, which allows for a systematic assessment of the accuracy of DFAs at different rungs of Jacob’s ladder^80^ without changing the model setup.^81^ The accessibility of higher-rung DFAs, such as HGGAs, is particularly important when adsorbing metal atoms on insulators and semiconductors, as there are very few experimental reference data on single-atom and nanocluster adsorption structures and energetics for these systems.

In this work, embedded cluster models are developed to study the adsorption of single metal atoms on oxygen-terminated diamond (110) surfaces. Starting from an idealized oxygen-terminated (110) surface, we build several models of surface oxygen vacancies and charged boron substitution defects and study the adsorption of gold atoms on these different systems. We use the embedded cluster models to perform a comprehensive benchmark of various state-of-the-art DFAs, combined with long-range dispersion correction methods, to assess their accuracy when predicting the adsorption structure and energetics of single gold atoms. A subset of the most accurate DFAs are used to study the diffusion barrier of the gold atoms on defective, doped, and idealized diamond surfaces. The results show that the thermally stable deposition of individual gold atoms on BDD requires the presence of surface vacancies or charged substitutional defects.

## Methods

Throughout the manuscript, we use the notation “χ^+ψ^/ϕ” to denote specific hybrid QM/MM methods, where χ is the DFA and ψ is the long-range dispersion correction used to describe the QM region, and ϕ is the force field used to describe the classical MM embedding region.

### Construction of QM/MM Embedded Cluster Models

**Figure 1:**
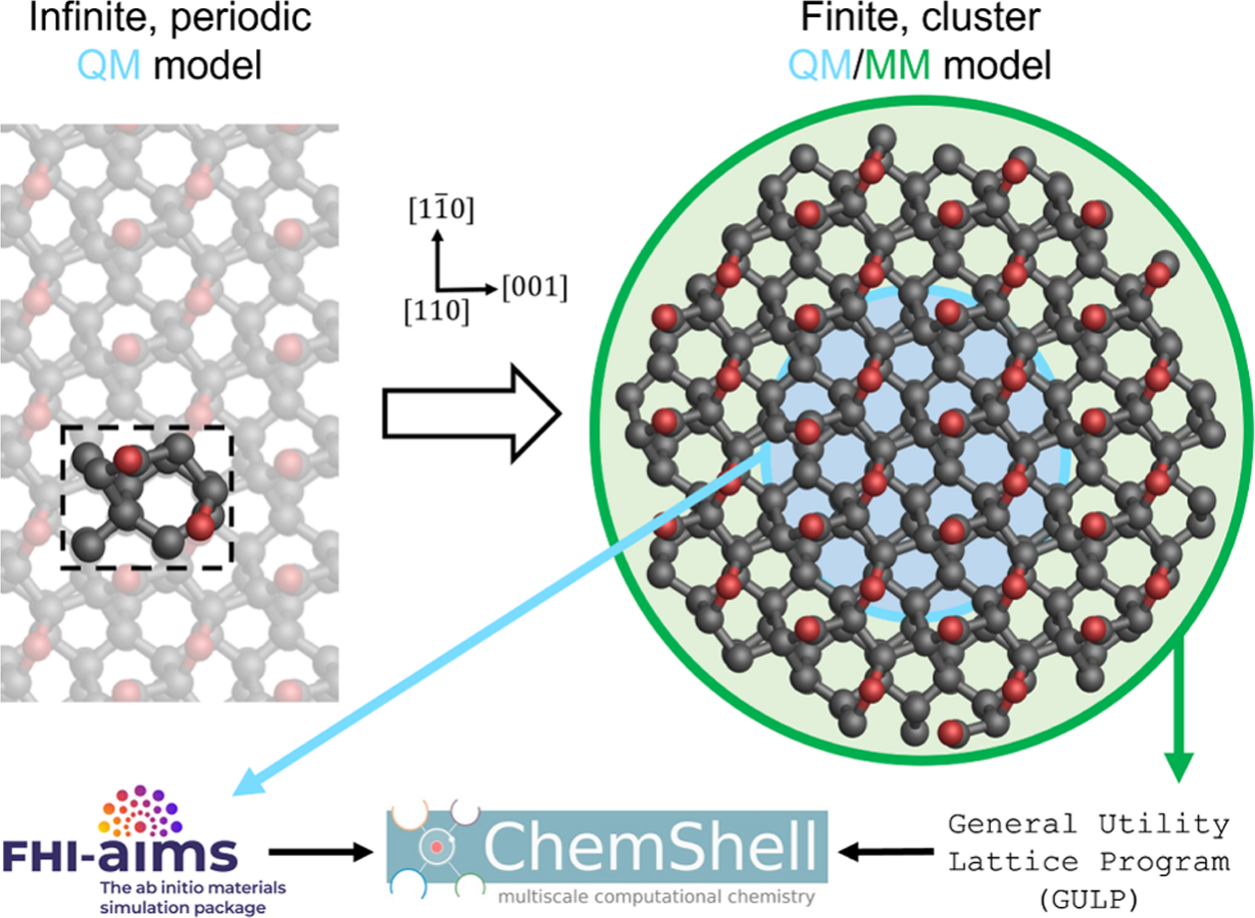
Process of converting an infinite, periodic surface model into a finite, embedded cluster model, including partitioning into quantum mechanical (QM) and molecular mechanical (MM) regions. Atoms within the blue circle represent the QM region of the cluster, while the green annulus represents atoms within the MM region. Also shown are the software packages used to treat the different regions. The surface is visualized from the [110] direction, with surface axes presented, and the unit cell outlines are shown with black dashed lines. Carbon and oxygen atoms are colored gray and red, respectively.

The Py-ChemShell^79,82,83^ software package is used to cut hemispherical clusters of radius 20.0 *a*_0_ (and active radius 10.0 *a*_0_) from the PBE^+TS^-optimized periodic models of the surface. Figure 1 details the cutting and partitioning processes necessary to convert a periodic surface model into an embedded cluster with QM and MM regions. The FHI-aims^84^ and GULP^85,86^ software packages are used to treat the QM and MM regions, respectively. The FHI-aims electronic structure package enables highly efficient computation of both periodic and aperiodic systems within the same numerical framework,^81^ allowing for direct comparisons to be made. QM/MM energies are calculated using an additive scheme^87^ and the hydrogen link-atom approach^88^ is used to treat cleaved covalent interactions across the QM–MM interface, both as implemented within the Py-ChemShell^79,83^ software. The connect_toler keyword, which is a rescaling coefficient for van der Waals (vdW) radii to determine bonding interactions, was set to a value of 1.3 for all QM/MM calculations to ensure the correct hydrogen saturation of the QM region for the FHI-aims calculation. To ensure the numerical parameters for the embedded cluster were fully converged, the properties of the periodic slab model were compared to clusters with varying sizes of QM region; a QM region with 90 atoms was chosen after comparing the band gaps, root-mean-square deviations of atomic positions, and single gold atom adsorption energetics. The cluster parametrization was performed with the PBE^+TS^/REBO method (where PBE^89^ is the density functional approximation, “TS” refers to the pairwise, long-range Tkatchenko–Scheffler (TS)^90^ dispersion correction method, and “REBO” is the reactive empirical bond order potential^91,92^), and further details of the convergence study are given in Figure S1 in the Supporting Information (SI). A comparison of the computational performance of periodic and embedded cluster models is shown in Figure S2, showcasing the significant computational gains from using the QM/MM approach compared with the periodic surface slab model.

### Construction of Structures

For most electrochemical applications, polycrystalline diamond is used. The diamond electrode is commonly grown via chemical vapor deposition (CVD),^93,94^ as opposed to high-pressure, high-temperature^95^ synthesis. Depending on CVD growth conditions, the (110) facet typically grows faster than the (111) and (100) facets.^96−98^ In polycrystalline samples, which are typically cheaper to grow than single-crystal samples for large-area technological applications, the (110) facets can be revealed by mechanically polishing to a surface roughness where the surface is predominantly (110)-textured,^99^ as has been experimentally demonstrated using electron backscatter diffraction^100^ and STEM.^36^ For electrochemical applications, which is the context of this work, the polycrystalline diamond material can thus be treated as a textured surface with a dominant (110) orientation.^99^

**Figure 2:**
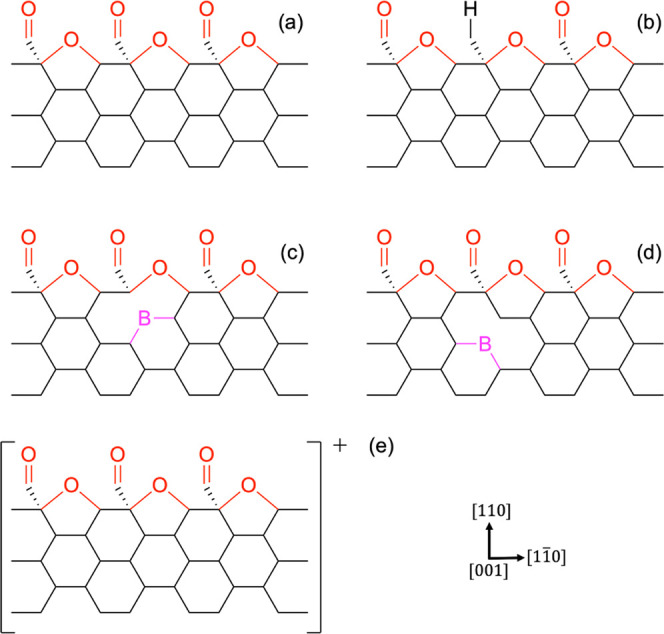
Skeletal visualizations of the substrate models investigated. Substrates are (a) a pristine oxygen-terminated diamond (110) surface, (b) a saturated carbonyl oxygen vacancy (SCOV)-defective surface, (c) a pristine surface with a boron dopant in the second layer, (d) a pristine surface with a boron dopant in the third layer, and (e) a pristine surface with a delocalized triel (group 13 element) dopant. Visualizations are shown from the [001] direction.

After CVD synthesis, the polycrystalline diamond surfaces are polished and chemically processed with strong oxidizing agents, rendering them oxygen-terminated.^99^ The fully oxygen-terminated surface model in Figure 2a represents the idealized surface and forms the starting point of the current study. The surface termination of the (110) surface was recently characterized in a joint computational-experimental study as dominated by coexistent and adjacent carbonyl and ether groups when synthesized via CVD.^99^ The experimental polycrystalline surfaces will likely exhibit coverage limitations at ambient conditions, though the proposed model is consistent with infrared and X-ray photoelectron spectroscopy measurements.^99,101−104^

Surface defects and impurities influence the properties for chemical applications, with oxygen vacancies in metal oxides previously shown to affect the catalytic properties of small gold clusters.^37,105−107^ Thus, several different point defects are explored in our work. A point defect at the surface is modeled by removing a single carbonyl oxygen, as shown in Figure 2b. To ensure the defect is modeled correctly, a PBE^+TS^/REBO structure optimization was performed after the removal of the carbonyl oxygen atom; as diamond surfaces are usually hydrogen-terminated after CVD growth,^93^ the uncoordinated carbon atoms are subsequently saturated with hydrogen atoms and the surface was reoptimized using PBE^+TS^/REBO. The defect is referenced as a saturated carbonyl oxygen vacancy (SCOV) herein.

Boron-doping is commonly used in electrochemical applications,^36,56^ and thus we also investigated the effect of boron dopants at the surface of the oxygen-terminated diamond (110) surface. The boron dopant can be situated at the surface or deep within the bulk BDD. To model the surface case, where the effects of the boron are localized, a boron atom is explicitly introduced to replace a carbon atom within the QM region of the QM/MM embedded cluster model, positioned in the second and third carbon layers of the surface, as shown in Figure 2c,d, respectively. The explicit presence of the boron atom in the surface layers is presumed to not affect the long-range structure or stability of the oxygen termination on the substrate surface.

To model boron dopants located deep within the bulk BDD, where the effects of the dopant are delocalized, a formal charge of +1 *e* is placed on the entire QM region to account for the effective loss of one electron in the system, as shown in Figure 2e. The model with a delocalized charge is not boron-specific, as boron is not explicitly included and thus is applicable for any delocalized single substitutional triel (group 13 element), such as aluminum, gallium, or indium. These non-boron triels are not common diamond dopants, but example realizations include aluminum dopants that induce superconductivity,^108,109^ though boron was deemed to be a better dopant to attain superconductivity;^109^ gallium dopants that suppress the graphitization of diamond tools by increasing their wear resistance;^110,111^ and indium dopants that improve the wettability of diamond.^112^ In our models, the effect of a single dopant atom was included within the QM region to match common boron dopant densities.^55^ All structures were constructed with the Atomic Simulation Environment^113^ Python package.

### Computational Settings

QM DFT^68,69^ calculations were performed using the all-electron numeric atomic orbital FHI-aims^84,114−119^ code. All calculations were performed with standard default “tight” basis set definitions (2020 version). The following convergence criteria were set for all FHI-aims self-consistent field calculations: 1 × 10^–6^ eV for the total energy, 1 × 10^–2^ eV for the sum of eigenvalues, 1 × 10^–5^ e/*a*_0_^3^ for the charge density, and 1 × 10^–4^ eV Å^–1^ for the energy derivatives. A criterion of 1 × 10^–2^ eV Å^–1^ for the maximum residual force component per atom was applied for the structure optimization calculations. Spin polarization was accounted for in all calculations, with the initial spin moment on the gold atom set to 1 to account for the single unpaired electron and its doublet ground state, and scalar relativistic effects were included via the atomic zero-order regular approximation.^84^

Unless otherwise specified, the TS^90^ dispersion correction method is used to account for vdW interactions in calculations with GGAs and HGGAs. The TS^90^ method was not used alongside MGGAs, which already account for a certain level of midrange interactions,^120^ or local density approximation DFAs (LDAs), which exhibit an artificial energy minimum between subsystems that can be mistaken for vdW stabilization.^71^ For periodic calculations, the interaction between the gold atom and its periodic images is excluded for the TS dispersion correction. Additional calculations were performed using MBD schemes, specifically, the range-separated self-consistently screened (MBD@rsSCS)^76^ and non-local (MBD-NL)^77^ variants; the choice of dispersion correction is indicated where considered.

The PBE^89^ GGA is the primary DFA used herein, though several other DFAs are considered. As the embedded cluster model is constructed with the PBE^+TS^ optimized surface model, DFAs were chosen for comparison when the diamond lattice constants are within ±0.02 Å of the PBE^+TS^ value. The filtering of DFAs ensures interatomic distances within the diamond substrate are not artificially strained when applying DFAs, allowing accurate comparisons to be made between DFAs. Lattice constant values for DFAs were either taken from the Materials Science and Engineering data set^121^ or, for DFAs not included within the data set, were calculated by optimizing the lattice vectors of the primitive diamond unit cell with a two-atom motif. The DFAs considered are implemented within FHI-aims or available via an interface to the Libxc^122^ library, and represent different rungs of Jacob’s ladder.^80^ The LDAs investigated are GDSMFB,^123^ KSDT^124^ and PZ-LDA;^125,126^ the GGAs studied are PBE,^89^ PBEsol,^127^ revPBE,^128^ and RPBE;^129^ and the MGGAs examined are SCAN,^130^ rSCAN,^131^ M06-L,^132^ TPSS,^120^ TPSSloc,^133^ and revTPSS.^134^ The following HGGAs are also considered: HSE03,^135^ HSE06,^136^ PBE0^137^ and PBEsol0.^138^ The dfauto(139) implementation within FHI-aims^84^ was used to run calculations with the SCAN^130^ and rSCAN^131^ MGGAs, and the standard screening parameter of 0.11 *a*_0_^–1^ was set for the HSE06^136^ HGGA.

MM calculations were performed with the GULP^85,86^ software package. The REBO potential^91,92^ was used to run MM calculations as it accurately describes hydrocarbon–oxygen interactions^92^ and predicts carbon–carbon bond lengths and angles within diamond.^91^ Comparative calculations were also performed using the Tersoff^140^ force field to benchmark against the REBO potential, confirming the suitability of the latter for our work; the results of these calculations are given in Section S3 in the SI.

Using a Mulliken analysis,^141^ density of states graphs were plotted via the logsdail/carmm(142) GitHub repository, with a Gaussian broadening value of 0.02 eV used for smoothing.

### Energy Calculations

The adsorption energy, *E*_ads_, of a single gold atom can be calculated as
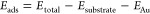
1where *E*_total_ is the total energy of the gold–diamond complex, *E*_substrate_ is the energy of the clean surface onto which the gold cluster was adsorbed, and *E*_Au_ is the energy of the isolated gold atom.

For structure optimizations with any QM/MM method, the active region of the PBE^+TS^/REBO-optimized oxygen-terminated diamond substrate was reoptimized using the respective DFA and force field combination. A single gold atom was then placed 1.5 Å above the adsorption site, and reoptimization was conducted using the specified QM/MM method. For the construction of binding energy curves using a specified QM/MM method, single-point calculations were performed on the specified QM/MM-optimized surface substrate with the gold atom being placed at various heights above the surface.

To assess the stability of the gold adatom in its adsorption site at finite temperatures with a specified QM/MM method, the gold atom was first translated to a new site along either the [001] or the [11̅0] directions and placed 1.5 Å above the specified QM/MM-optimized surface. A constrained optimization was then conducted, where the position of the gold atom was only allowed to relax along the [110] direction, with motion along the [001] and [11̅0] directions frozen. The thermal stability of the gold atom with any specified QM/MM method was then calculated as the energy difference, Δ*E*, between the stable equilibrium structure and the highest-energy structure along the constrained path.

## Results and Discussion

### Effect of Defects and Dopants

**Figure 3:**
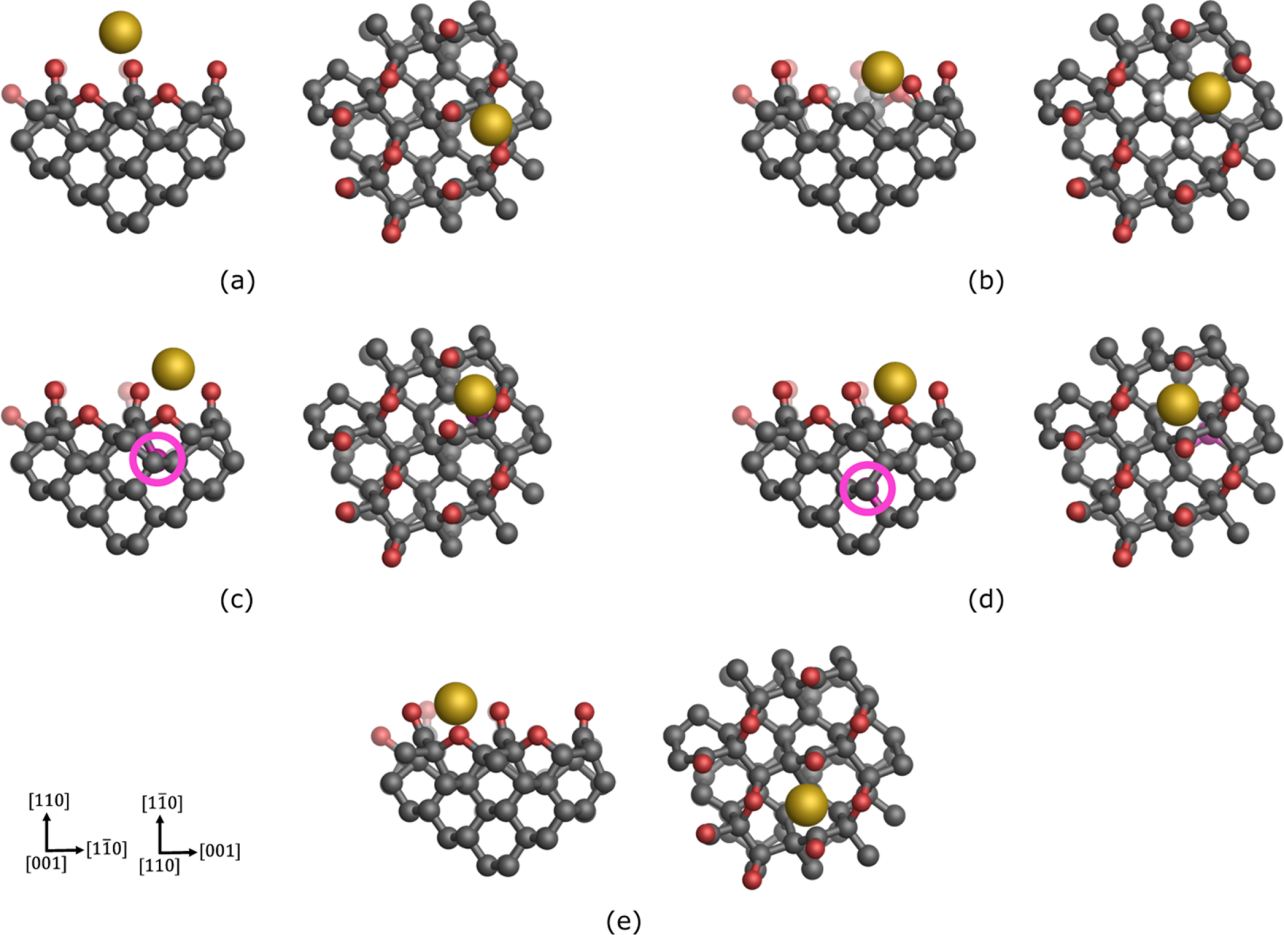
Orthographic ball-and-stick visualizations of a gold adatom on different substrate models as optimized using the PBE^+TS^/REBO method. Substrates are (a) a pristine oxygen-terminated diamond (110) surface, (b) a defective surface with a saturated carbonyl oxygen vacancy (SCOV), (c) a boron-doped surface with the dopant in the second layer, (d) a boron-doped surface with the dopant in the third layer, and (e) a delocalized triel-doped surface. Visualizations of the quantum mechanical (QM) region are shown from the [001] and [110] directions, and surface axes are also shown, with the saturating hydrogen species at the QM region boundary excluded for clarity. Carbon, oxygen, hydrogen, boron, and gold atoms are shown in gray, red, white, pink, and gold, respectively. For clarity, pink circles are included to show which carbon atom the boron atom is situated behind for (c) and (d).

The pristine, fully oxygen-terminated diamond (110) surface was used as the starting point for all QM/MM models, as shown in Figure 3a. Other systems were also studied, where defects and dopants were introduced into the surface model, namely, a SCOV-defective surface, which is visualized in Figure 3b, and boron-doped surfaces with the dopant modeled explicitly and implicitly, which are visualized in Figure 3c–e. The interactions between the gold atom and each surface are discussed in more detail below. In all cases, different adsorption sites were explored to identify the most stable lateral sites.

Table 1 summarizes the adsorption energy, adsorption structure, and the Mulliken charge^141^ of the single gold atom atop these surfaces. The introduction of defects or dopants into the idealized surface seems to strengthen the adsorption energy of the gold atom, which is reflected in the lower adsorption height, indicating the closer proximity of the adatom to the surface. For all investigated defective and doped surfaces, the sign of the Mulliken charge^141^ on the gold atom was positive, which is indicative of charge transfer from the gold atom into the surface and explains the relatively higher adsorption energies. In contrast, for the pristine surface, the Mulliken charge is negative, indicating a charge accumulation. It should be noted that the more complete a basis set is, the more ambiguous a Mulliken analysis becomes as it is not *a priori* clear which electrons should be counted toward the basis functions of one atom rather than another. We use Mulliken analysis only as a qualitative indicator to identify trends across the systems.

**Table 1:** Adsorption Energies, Adsorption Heights, and Mulliken Charges for a Single Gold Adatom on Various Oxygen-Terminated Diamond (110) Surface Substrates^a^

system	adsorption energy (eV)	adsorption height (Å)	Mulliken charge (*e*)
pristine	–0.30	1.71	–0.14
SCOV	–2.31	–0.12	+0.07
boron dopant (2nd layer)	–1.66	1.03	+0.28
boron dopant (3rd layer)	–1.75	0.35	+0.16
delocalized triel dopant	–1.98	0.36	+0.26

a Adsorption energies were calculated by using the PBE+TS/REBO method, and adsorption heights are given with respect to the averaged plane of carbonyl oxygen atoms.

#### Pristine Surface

In the case of the idealized, fully oxygen-terminated surface, the gold adatom weakly adsorbs onto a carbonyl oxygen atom at a height of 1.71 Å above the surface, with an adsorption energy of −0.30 eV, as detailed in Table 1. The weak adsorption of the gold adatom on the pristine surface is expected, due to the high stability of the coexistent carbonyl and ether functional groups on the diamond surface.^99^ The valencies of all surface atoms are satisfied;^99^ thus, there are no unpaired electrons for the gold atom to interact with, which means the interaction between the adatom and the surface is governed by weak long-range interactions such as vdW forces and electrostatics.

#### SCOV Defect

As depicted in Figure 3b, the gold adatom adsorbs significantly closer to the SCOV-defective diamond surface than for the pristine surface, with a stronger adsorption energy of −2.31 eV, indicating that this is a much more stable adsorption complex. Indeed, a negative adsorption height is observed, as shown in Table 1, which indicates that the gold atom sits below the plane of carbonyl oxygen atoms and is thus much closer to the surface carbon atoms than in the pristine surface. This phenomenon occurs as one of the C–O bonds within a surface ether group breaks, and the gold atom is inserted to form a C–Au–O–C arrangement.

**Figure 4:**
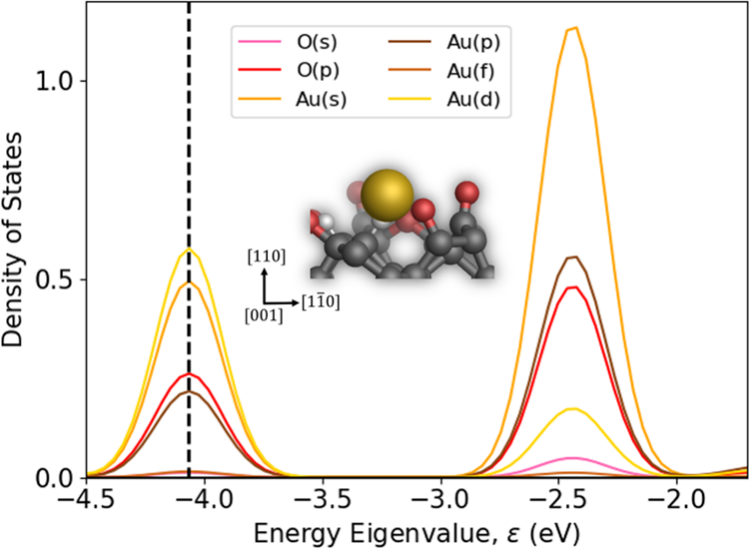
Projected density of states of the orbital contributions from a single gold (Au) atom and its neighboring former-ether oxygen (O) atom on an oxygen-terminated diamond (110) surface with a saturated carbonyl oxygen vacancy (SCOV) defect after optimization with the PBE^+TS^/REBO method. The black dashed vertical line indicates the position of the highest occupied molecular orbital. Also shown is an orthographic ball-and-stick visualization of a single gold adsorbed onto the SCOV-defective surface along the [001] direction. Carbon, oxygen, hydrogen, and gold atoms are shown in gray, red, white, and gold, respectively.

To elucidate the nature of the bond between the gold adatom and the diamond surface, the projected density of states of the gold atom and its neighboring former-ether oxygen atom was computed based on a Mulliken analysis^141^ and is shown in Figure 4. The highest occupied molecular orbital (HOMO) is shown by the peak centered at an eigenenergy of −4.1 eV and includes contributions from oxygen p-states as well as gold s-, p-, and d-states. In contrast, the lowest unoccupied molecular orbital (LUMO), which is shown by the peak centered at −2.4 eV, is dominated by gold *s*-states with contributions from both oxygen and gold p-states and a small contribution from gold d-states. In the HOMO and LUMO peaks, the contributions from oxygen s- and gold f-states are near-zero and negligible. The presence of the single gold atom can therefore be seen to form both bonding and antibonding orbitals and is indicative of a bonding interaction between spd-hybridized orbitals of the gold atom and the oxygen p orbitals, which agrees with previous observations for interactions between gold and oxygen atoms.^143^

The Au–O bond length on the SCOV-defective surface is 2.09 Å, which is only 0.07 Å longer than the sum (2.02 Å) of the covalent radii for gold (1.36 Å) and oxygen (0.66 Å),^144^ while a similar bond length (2.06 Å) has been observed in gold-based trifluoromethoxy complexes.^145^ As shown in Table 1, the positive sign of the Mulliken charge^141^ on the gold atom is indicative of a loss of electron density from the gold atom to the surface. In contrast, the formerly ether oxygen atom has a Mulliken charge^141^ of −0.30*e*, which indicates charge accumulation. The effective valence charge, which is the difference between the formal and Mulliken charges of the anion, can be used as a measure of ionic/covalent character.^146^ An effective valence charge of 0 *e* would indicate a dominantly ionic character of the bond while larger values would indicate increasing levels of covalency.^146^ If the Au–O bond is assumed to be ionic (i.e., Au^+^ O^–^), then the formal charge of the oxygen anion would be −1 *e*, which would result in an effective valence charge of 0.70*e*. Monovalent ionic compounds such as sodium halides were evaluated to have effective valence charges less than 0.6*e*,^146^ which would suggest that the interaction between the gold and the former-ether oxygen atoms is more ionic than covalent. As mentioned, an assumption was made by treating the Au–O bond as ionic for the calculation of the effective valence charge, while Mulliken charge decompositions have inherent issues of their own, as discussed above. The analysis indicates that the interaction has attributes of a polar covalent bond and an ionic bond, rather than a nonpolar covalent bond, which is expected given the greater electronegativity of oxygen with respect to gold.^147^

#### Single Substitutional Boron Dopant

The boron-doped systems result in single gold atom adsorption that is stronger than that for the idealized system, though not as strong as that of the SCOV-defective system (Table 1). The increased stability of the gold adatom in the presence of the boron dopant is expected because similar effects have been reported for the adsorption energy of hydrogen^148−151^ and metal atoms such as calcium^152,153^ and sodium.^154,155^ The stronger adsorption for boron-doped surfaces, as opposed to the undoped pristine surface, occurs as boron dopants possess one fewer valence electrons than the carbon atoms in diamond. Such p-type dopants form an electron-deficient region that the gold adatom is attracted toward.^154^ While the difference between the adsorption energies for the localized cases is slight at only 0.09 eV, the 0.68 Å difference in the adsorption height is more significant. The disparity in the adsorption heights is due to the location of the boron dopant within the surface layers. In the model where the dopant is in the second layer, the boron atom lies below an ether oxygen atom, whereas the boron dopant within the third layer lies below a carbonyl oxygen atom (see Figure 2). The gold atom is attracted to the electron-deficient regions caused by p-type dopants such as boron;^154^ in both cases, the gold atom adsorbs above the ether and carbonyl oxygen atoms that lie atop the second- and third-layer dopants, respectively, as shown in Figure 3c,d, respectively.

The adsorption energy and height calculated from the delocalized model, where a formal charge of +1 *e* was placed on the system, do not differ significantly from the model with the boron atom in the third layer, representing a localized charge defect (Table 1); the adsorption energy and height differ by only 0.23 eV and 0.01 Å, respectively. The similarity is expected, as the localized dopant has a more long-range, delocalized effect when it sits deeper within the surface. Unlike the pristine surface, the charge introduced in the delocalized model causes the structure of the surface atoms to change to accommodate the gold atom; the surface rearrangement means the gold atom is close to an ether oxygen atom and positioned between two carbonyl oxygen atoms, resulting in a smaller adsorption height and larger adsorption energy than for the pristine surface.

In general, the pristine, fully oxygenated diamond (110) surface exhibits weak adsorption of the gold atom. The introduction of defects or dopants into the surface significantly increases the adsorption energy of the gold atom; in particular, the SCOV defect results in a large adsorption energy of 2.31 eV. Projection of the density of states for the gold and neighboring carbon and oxygen atoms shows that the strong adsorption is due to the formation of a polar covalent bond between the gold adatom and the diamond surface. The introduction of boron dopants, both localized and delocalized, also increases the stability of the single gold atom on the surface compared to the pristine surface, although not to the same extent as for the SCOV defect.

### Assessment of Density Functional Approximations

Having established the surface structures that lead to more stable gold adsorption, we benchmarked the performance of different DFAs in order to confirm that the observed trends, as calculated above using PBE+TS, are retained irrespective of the DFA chosen. Different QM methods have been benchmarked for the pristine system, the SCOV-defective system, and the delocalized triel-doped system. The delocalized doped system was chosen particularly because: (i) with common boron dopant densities, the probability of finding the dopant atom far from the surface is much higher than finding it close to the top surface layers; (ii) the delocalized model is applicable to any triel dopant, not just boron; and (iii) the predicted adsorption height and energy of the adatom do not differ significantly from the case where the boron dopant in the third layer was used as a localized defect (see Table 1).

In addition to the DFA, the effects of the embedding force field environment and dispersion correction have been considered. The investigation details are provided in the SI; Table S1 shows that embedding the QM region within a Tersoff^140^ force field environment results in a change in the adsorption height of the gold atom by 0.05 Å compared to REBO^91,92^ for the idealized surface. Both force fields predict virtually identical adsorption energies, showing that the choice of embedding force field environment does not have a large effect on adsorption energetics.

Furthermore, the pairwise TS dispersion correction method^90^ was also benchmarked against the MBD@rsSCS^76^ and MBD-NL^77^ methods for the three aforementioned surfaces, with results presented in Table S2 and Figure S3 in the SI. Neglection of long-range dispersion interactions yields considerable underbinding of the adatoms, while all tested dispersion corrections yield closely similar adsorption energies and heights. Therefore, a long-range dispersion correction was included for all DFAs that do not account for mid/long-range dispersion interactions in their derivation, such as GGAs.^70,71^

**Figure 5:**
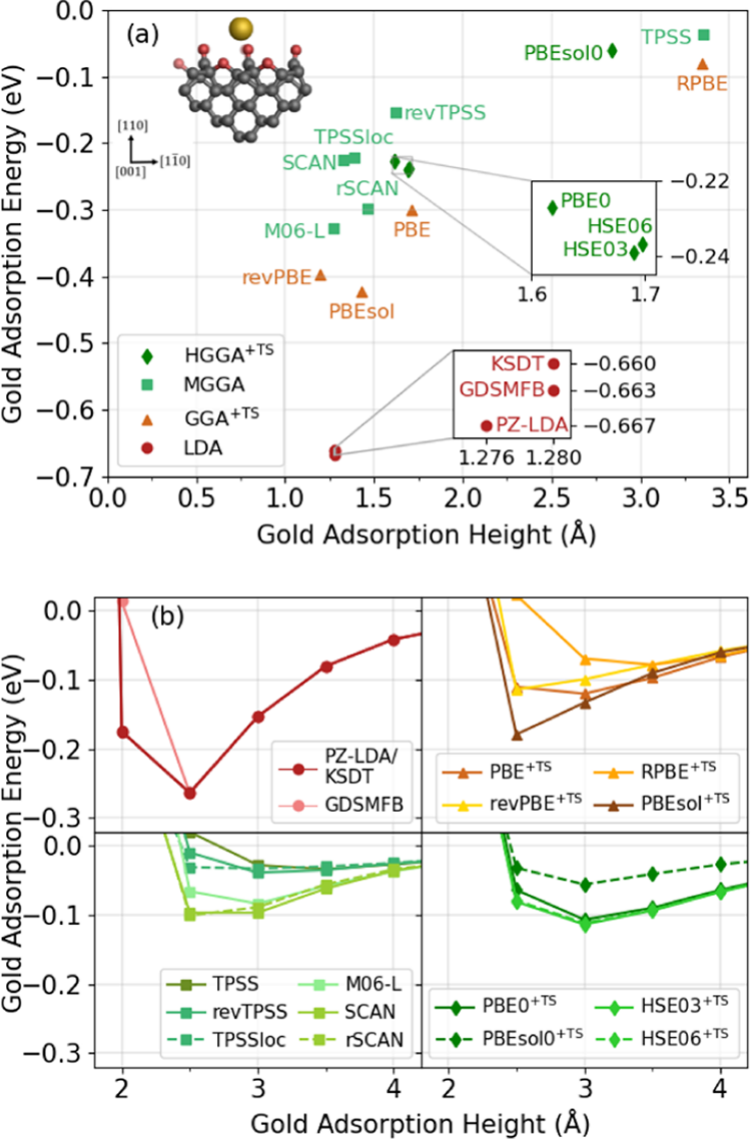
Plots benchmarking the performance of various density functional approximations for gold adatom adsorption on an idealized oxygen-terminated diamond (110) surface. (a) Scatter graph showing the adsorption energy and adsorption height of a single gold adatom after a full geometry optimization. (b) Unrelaxed binding energy curves showing the adsorption energy of a single gold adatom as a function of height above the substrate surface. In (b), density functional approximations are divided according to (from left to right) the following: local density approximations (LDAs), Tkatchenko–Scheffler (TS)-corrected generalized gradient approximations (GGAs), meta-GGAs (MGGAs), and hybrid GGAs (HGGAs).

**Figure 6:**
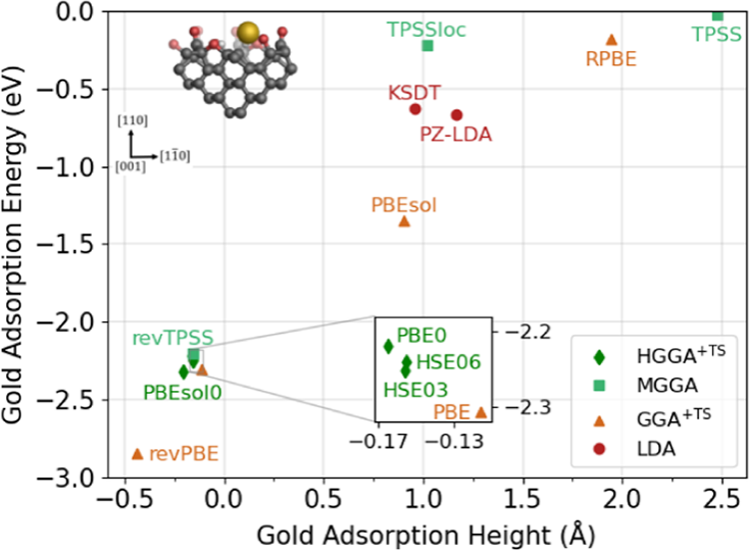
Scatter graph showing the adsorption energy and height of a single gold adatom after a full geometry optimization using various density functional approximations on an oxygen-terminated diamond (110) surface with a saturated carbonyl oxygen vacancy defect. Density functional approximations are identified according to their rung on Jacob’s ladder: local density approximations (LDAs), Tkatchenko–Scheffler (TS)-corrected generalized gradient approximations (GGAs), meta-GGAs (MGGAs), and TS-corrected hybrid GGAs (HGGAs).

**Figure 7:**
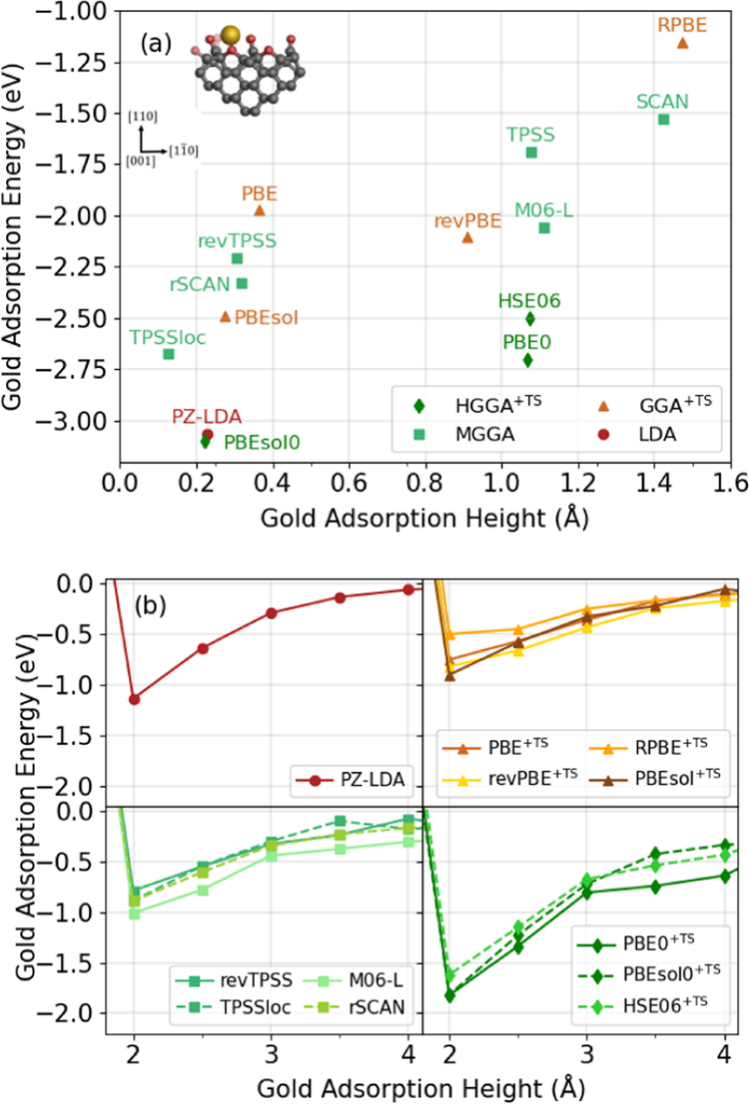
Plots benchmarking the performance of various density functional approximations on a delocalized triel-doped oxygen-terminated diamond (110) surface. (a) Scatter graph showing the adsorption energy and height of a single gold adatom after full geometry optimization. (b) Unrelaxed binding energy curves showing the adsorption energy of a single gold adatom as a function of height above the substrate surface. In (b), density functional approximations are divided according to (from left to right): local density approximations (LDAs), Tkatchenko–Scheffler (TS)-corrected generalized gradient approximations (GGAs), meta-GGAs (MGGAs), and TS-corrected hybrid GGAs (HGGAs).

The performance of the DFAs is benchmarked by comparing the adsorption energy and gold adatom height after a full QM/MM geometry optimization (Figures 5a, 6, and 7a). Furthermore, binding energy curves were constructed using a series of single-point QM/MM calculations, where the gold adatom was placed at various heights above the unperturbed pristine and defective surfaces (Figures 5b, S5, and 7b). The former allows investigation of how different DFAs predict short-distance bonding scenarios, while the binding energy curves provide information about the mid- to long-range interaction between the gold atom and the different surface substrates.

#### Pristine Surface

Figure 5a details the performance of various DFAs on a pristine surface after a full QM/REBO optimization. All DFAs predict weak adsorption of the single gold atom, with adsorption energies ranging from −0.04 to −0.67 eV. An inverse relationship can be seen between the adsorption height and the adsorption energy, which is expected as a smaller adsorption height is generally reflective of a chemical bond and stronger interaction between the adsorbate and substrate. The DFAs for each rung of Jacob’s ladder^80^ produce results that are generally grouped together in specific areas. LDAs (GDSMFB, KSDT, and PZ-LDA) predict the largest adsorption energy (between −0.66 and −0.67 eV). The result is in line with observations that LDAs typically overestimate the interaction at hybrid organic–inorganic interfaces,^70,71^ which results in overestimated adsorption energies and underestimated adsorption heights.^70,71^ Most TS-corrected GGAs, MGGAs, and TS-corrected HGGAs are also grouped together and generally predict adsorption energetics similar to those of PBE; the exceptions are the RPBE GGA, the TPSS MGGA, and the PBEsol0 HGGA, which all show weaker adsorption energetics.

For the GGAs, the differences in the adsorption energy (and height) are subtle. The revPBE GGA predicts stronger adsorption than PBE by only 0.1 eV (−0.40 as opposed to −0.30 eV). The result is expected as both PBE and revPBE possess the same mathematical form, as outlined in eq 2, for the exchange energy enhancement factor, *F*_*X*_:
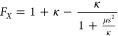
2where *s* is the reduced density gradient, and κ and μ are constants.^128^ The only difference between PBE and revPBE is that PBE specifies κ = 0.804, while revPBE softens this criterion to κ = 1.245.^128^ The PBEsol GGA only differs from the (rev)PBE formulation by reducing the *s*-dependence of *F*_*X*_ by reducing μ,^127^ and subsequently predicts a similar adsorption energy of −0.42 eV. The similarities between the PBE, revPBE, and PBEsol formulations for *F*_*X*_ indicate why these GGAs give fairly similar adsorption energetics. The RPBE GGA, however, possesses a different mathematical form for *F*_*X*_,^129^ and has been previously highlighted to not perform well for physisorbed systems where vdW effects govern adsorption,^156−158^ which helps to explain the disparity between results attained using RPBE and other PBE-like GGAs.

Most of the MGGAs predict adsorption energetics that are similar to each other and to most GGAs; the only exception is the TPSS MGGA, which predicts similar adsorption energetics to the RPBE GGA. Some DFAs have been developed to correct for the discrepancy between TPSS and GGAs by building TPSS-like MGGAs and “fitting” to GGA results.^133,134^ The TPSSloc MGGA uses a localized PBE-like DFA for the correlation within a TPSS-like DFA form,^133^ while the revTPSS formulation is based on the PBEsol modification to the PBE correlation.^134^ These changes to the TPSS formalism might explain why the TPSSloc and revTPSS results align better with GGA results than TPSS. The M06-L MGGA also includes the PBE exchange energy density within its formulation for the exchange energy,^132^ which might also explain its similar performance to PBE-derived DFAs. The slightly stronger adsorption energy for single gold atoms with M06-L, compared to PBE, has been previously observed for adsorption on Mg(100).^159^ Overall, all investigated MGGAs apart from TPSS can be seen to predict adsorption energetics similar to the PBE-predicted values.

The HSE03, HSE06, and PBE0 HGGAs predict similar adsorption energetics to all GGAs apart from RPBE. The PBEsol0 HGGA predicts much weaker adsorption than the other HGGAs, as well as relative to the PBEsol GGA that accounts for 75% of the exchange energy within PBEsol0.^138^ The result is somewhat surprising given the agreement seen between PBE-derived HGGAs but clearly mixing the exchange energy from PBEsol and Hartree–Fock components, as is done within PBEsol0,^138^ can lead to contrasting results (for this system at the very least). Furthermore, PBEsol0 was designed to provide more accurate structural and energetic predictions for solids than GGAs,^138^ and therefore may not perform as well for surface adsorption.

Moving onto the unrelaxed binding energy curves over the pristine surface, as shown in Figure 5b, all DFAs give a curve with an energy minimum between 2.5 and 3.5 Å height above the surface. The binding energies are based on restraining the gold atom at different heights above the clean surface structure, and therefore, the optimal adsorption heights differ from Figure 5a, which reports fully optimized structures. LDAs have an adsorption energy minimum of −0.26 eV at an adsorption height of 2.5 Å, which is closer to the surface than for other methods. A deeper energetic minimum is observed for the LDAs and is indicative of stronger binding, which is in line with observations that LDAs predict stronger adsorption.^70,71^ For the GGAs, the revPBE and PBEsol choices have binding energy minima of −0.11 and −0.18 eV, respectively, at 2.0 Å. The PBE binding energy minimum (−0.12 eV) lies between the revPBE and PBEsol values, though this value occurs at a larger adsorption height of 2.5 Å. The RPBE binding energy minimum is the shallowest of all GGA curves, with a value of −0.08 eV, and this minimum arises at the largest adsorption height of all investigated DFAs (3.5 Å), matching the results when geometry optimization.

For MGGAs, DFAs within the same families have similar binding energy curves. TPSSloc and revTPSS have adsorption energy minima of −0.03 and −0.04 eV, respectively, at an adsorption height of 3.0 Å. The adsorption height is the same as for PBE, but the adsorption energies are much smaller, which explains why these two MGGAs predict weaker adsorption than PBE in Figure 5a. TPSS has a similar adsorption energy minimum of −0.03 eV at an adsorption height of 3.5 Å, which is the same height as the RPBE GGA, albeit with a lower adsorption energy. The SCAN and rSCAN MGGAs have similar binding energy curves, with minima of −0.10 eV at 2.5 Å, which is a similar adsorption energy minimum to the PBE GGA and the same adsorption height as the revPBE and PBEsol GGAs; the trend is reflected by the positions of the SCAN and rSCAN data points in Figure 5a. M06-L has an adsorption energy minimum at −0.08 eV at an adsorption height of 3.0 Å, similar to those of the PBE, SCAN, and rSCAN DFAs. The HSE03, HSE06, and PBE0 HGGAs have very similar binding energy curves, with adsorption energy minima at −0.11 eV at an adsorption height of 3.0 Å. The close agreement of the binding energy curves explains why these HGGAs are so close together in Figure 5a. In contrast, PBEsol0 HGGA has a much shallower adsorption energy minimum of −0.06 eV at 3.0 Å.

Overall, most GGAs, MGGAs, and HGGAs predict very similar binding energy curves. In particular, the PBE, revPBE, SCAN, rSCAN, PBE0, HSE03, and HSE06 binding energy curves are very closely clustered. The result suggests that, for the pristine surface where the gold adatom is weakly physisorbed, the mid- to long-range interactions, as captured in the binding energy curves, are all very similar, except for LDAs. The result indicates that most common DFAs perform very similar for the weakly bound case and suggests that dispersion-corrected PBE is an appropriate choice.

#### SCOV-Defective Surface

The second substrate of interest was a surface with a SCOV defect. To ensure that the SCOV defect was accurately modeled, the conformational isomerism of the structure centered at the former-carbonyl carbon atom was studied, and the results are presented in Table S3. The PBE0, PBEsol0, HSE03, and HSE06 HGGAs result in an anticlinal conformation (rather than the expected synclinal conformation), as is shown by the Newman projection^160^ in Figure S4. The anticlinal conformation may be a local energy minimum and not the correct physical conformation for the surface after the removal of a carbonyl oxygen atom, as is explained in Section S5 in the SI. To validate the greater stability of the synclinal conformation, the final PBE^+TS^/REBO-optimized SCOV-defective structures were reoptimized by using the respective HGGA^+TS^/REBO method before further use. Table S4 shows that the synclinal conformation is 0.73–0.86 eV more stable than the anticlinal conformation, depending on the HGGA used, confirming the metastable nature of the anticlinal minima identified with the HGGAs.

Figure 6 details the performance of various DFAs on an SCOV-defective surface. The introduction of a SCOV defect at the surface significantly increases the range of adsorption energies and heights compared to the idealized surface. The range of adsorption energy values indicate that DFAs such as PBE, revPBE, revTPSS, and the HGGAs predict much stronger adsorption and a possible bonding interaction between the gold adatom and the substrate surface. Both LDAs (PZ-LDA and KSDT) predict similar adsorption energies of −0.67 and −0.63 eV, respectively; however, there is quite a large range of adsorption energies predicted among TS-corrected GGAs, and all GGAs apart from RPBE predict stronger adsorption than the LDAs. The revPBE and PBE GGAs predict very strong adsorption (−2.84 and −2.31 eV, respectively). The negative adsorption heights mean that the gold adatom sits below the plane of carbonyl oxygen atoms, i.e., within the “well” caused by the vacancy. The PBEsol GGA predicts weaker adsorption than revPBE and PBE, but strong adsorption nonetheless with an adsorption energy of −1.35 eV. Much like in the case of the pristine surface, the RPBE GGA predicts a weak adsorption energy of −0.18 eV and predicts the gold adatom to adsorb 1.94 Å above the surface.

MGGAs predict a wide range of adsorption energies, much like the GGAs. The revTPSS MGGA predicts an adsorption energy of −2.20 eV, which is slightly weaker than that of the PBE GGA. The negative adsorption height indicates that revTPSS also predicts the gold adatom to sit below the plane of carbonyl oxygen atoms. TPSS, in contrast, predicts an adsorption energy of −0.03 eV, with the gold adatom adsorbing 2.48 Å above the surface, much like the RPBE GGA. The performance of TPSSloc differs quite a lot from adsorption on the pristine surface, with the MGGA predicting an adsorption energy of −0.22 eV, although the adsorption is closer to the surface than by RPBE and TPSS, with an adsorption height of 1.02 Å. The four investigated HGGAs predict strong adsorption of the gold atom, and the optimized adsorption heights and energies are very similar to the values predicted by revTPSS and PBE, as can be seen in Figure 6. While binding energy curves attained using the unrelaxed SCOV-defective surface do not directly correspond to the fully relaxed surface due to the significant amount of surface reconstruction upon the addition of a gold adatom, Figure S5 shows that even for the unrelaxed surface, the HGGA unrelaxed binding energy curves are very similar to the PBE curves. This suggests that for the SCOV-defective surface, where the gold atom is strongly chemisorbed, dispersion-corrected PBE again remains an appropriate DFA choice.

#### Delocalized Triel-Doped Surface

Figure 7 details the performance of various DFAs on the final substrate considered, which was a delocalized triel-doped surface. As can be seen in Figure 7a, the introduction of a charge into the surface significantly increases the adsorption strength compared to the idealized surface, with adsorption energies ranging from −1.16 to −2.84 eV. There is a general inverse relationship between the adsorption heights and energies, though DFAs are generally grouped into two areas of adsorption heights: 0–0.4 and 0.9–1.5 Å above the plane of carbonyl oxygen atoms. In the set of lower adsorption heights (0–0.4 Å), the surface atoms rearrange to accommodate the gold atom, and the gold atom gets closer to an ether oxygen atom and is positioned between two carbonyl oxygen atoms, resulting in a smaller adsorption height and a larger adsorption energy. In contrast, in the set of higher adsorption heights (0.9–1.5 Å), the surface does not change as much and sterically hinders the gold atom from getting closer to the ether oxygen atom. The gold atom therefore binds to the carbonyl oxygen atom, resulting in a larger adsorption height and a weaker adsorption energy.

As shown in Figure 7a, revPBE (−2.11 eV) predicts stronger adsorption than PBE, while PBEsol (−2.49 eV) predicts slightly stronger adsorption than both PBE and revPBE. MGGAs also generally predict similar adsorption energies to GGAs, with some exceptions. The SCAN MGGA predicts the second-weakest adsorption (−1.53 eV) of all investigated DFAs and has the second-largest adsorption height of 1.43 Å, which is not too dissimilar to the RPBE-predicted adsorption height. The TPSS and M06-L MGGAs predict stronger adsorption than both RPBE and SCAN, while both TPSS and M06-L predict similar adsorption heights to PBE0 and HSE06, but predict weaker adsorption energies. In contrast, revised versions of TPSS and SCAN, namely, revTPSS, TPSSloc, and rSCAN, generally predict stronger adsorption energies of −2.21, – 2.68, and −2.33 eV, respectively, with the gold atom adsorbing much closer to the surface. The similarity between the revised MGGAs and the PBE-based GGAs can be attributed to their GGA-based formulation. As discussed earlier, TPSSloc includes a PBE-like component,^133^ while revTPSS is based on the PBEsol modification to PBE.^134^

Unlike the pristine and SCOV-defective surfaces, HGGAs generally predict stronger adsorption than GGAs and MGGAs on the triel-doped surface. PBEsol0 predicts adsorption energetics very similar to that of PZ-LDA, with the strongest adsorption energy of all investigated DFAs (−3.10 eV) and a very small adsorption height of 0.22 Å, which is only 0.01 Å lower than the PZ-LDA-predicted value. Despite predicting stronger adsorption energies, PBEsol0 predicts a similar adsorption height for the single gold atom compared to the aforementioned revised MGGAs, PBE, and the PBEsol GGA on which PBEsol0 is built. In contrast, the PBE0 and HSE06 results differ a fair amount from the PBE result, despite both HGGAs being built upon PBE components within their formulations. The results indicate that GGAs and MGGAs may not fully capture the mid- and long-range interactions between the gold atom and surface, whereas HGGAs such as HSE06 and PBE0 do, potentially rendering them more appropriate DFAs than (M)GGAs for the description of adsorption at charged defects. That being the case, all investigated DFAs still predict stronger adsorption of the gold atom on the triel-doped surface than on the idealized surface, which is consistent with the adsorption trends seen with PBE and observed in Table 1.

As can be seen in Figure 7b, the unrelaxed binding energy curves calculated using LDAs, GGAs, and MGGAs are very similar, and there are also only small deviations between GGAs and between MGGAs. The HGGA binding energy curves have deeper minima than those of the lower-rung DFAs, suggesting a stronger attraction between the gold adatom and the surface. The results indicate that the choice of DFA (within a rung) does not strongly affect the binding energy curves in the mid- and long-range, which suggests that classical electrostatic interactions between the charged defect and the polarizable gold adatom are the dominant contribution.

In summary, for the idealized and SCOV-defective surfaces, the PBE prediction is consistent with higher-rung MGGAs and HGGAs, accurately capturing the physisorption and chemisorption of the gold adatom, respectively. Good agreement was also observed with most other GGAs, as well as many higher-rung MGGAs and HGGAs. The consistency in observations is important, as there are no existing experimental data to describe the adsorption energetics of single gold atoms on such surfaces. Some disagreement, however, was observed between PBE and higher-rung HGGAs for the delocalized triel-doped surface. The differences between PBE and HGGAs indicate that PBE is perhaps not the most appropriate DFA to treat charged defects, although PBE was still able to capture the fact that the adsorption is stronger on the charged defect compared to the pristine surface. Most importantly, the adsorption trends observed in Table 1 between pristine, defective, and doped surfaces are robust with respect to the choice of embedding force field, dispersion correction scheme, and DFA.

### Thermal Stability of Deposited Single Gold Atoms

Having established how the adsorption energy and height of a single gold atom vary when adsorbed at oxygen-terminated diamond (110) surfaces with different defects and dopants, we turn our attention to the thermal stability of the atom in its adsorption site. Using identical-location STEM, Hussein et al. observed gold atoms to be very stable atop polycrystalline BDD surfaces. Before transfer to the microscope, samples undergo thermal baking,^36^ yet single adatoms can be observed. Also, the momentum transfer from the highly energetic electron beam (∼200 kV) is significant, yet little to no movement of the gold atoms is observed on BDD over multiple measurements of the same image area.^36^ This suggests that significant energy barriers need to be overcome for the gold atom to leave its adsorption site. However, the barriers for diffusion of a single gold atom on pristine oxygen-terminated BDD were previously calculated with PBE and found to be too low^36^ (*vide infra*) to withstand the above processes. These previous findings suggest that the high stability of single gold atoms observed by Hussein et al.^36^ is likely due to surface defects and (boron) dopants that were not visible within their microscopy images. To investigate the hypothesis, we performed constrained QM/REBO optimizations to construct minimum energy paths for the lateral motion of a single gold atom across the pristine, SCOV-defective, and explicitly boron-doped surfaces after adsorption.

**Figure 8:**
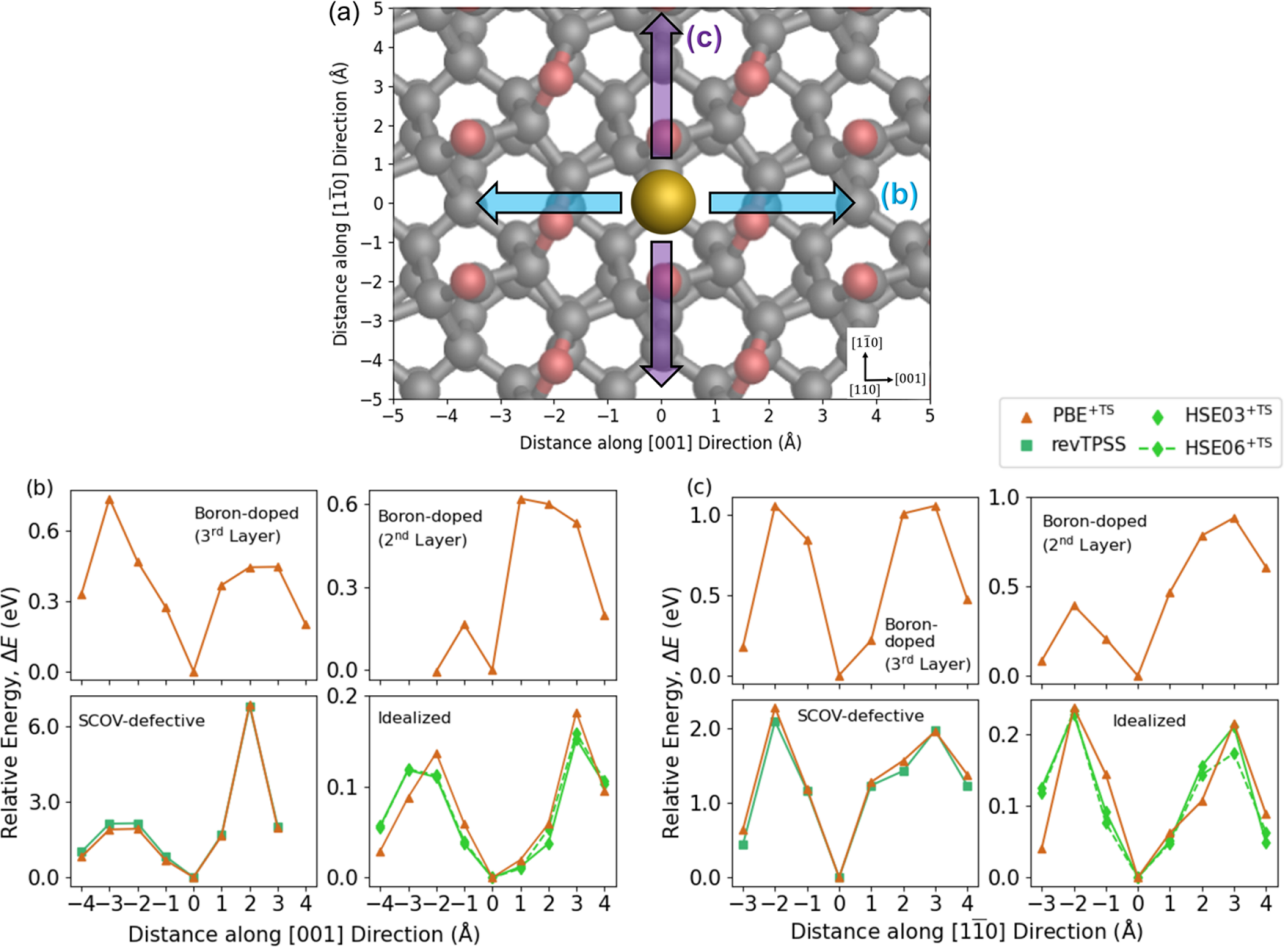
Relative energies (Δ*E*) of translating a single gold atom across various oxygen-terminated diamond (110) surface substrates. The initial adsorption site is placed at the origin on each graph. (a) Paths of motion along the idealized surface; (b) relative energies along the [001] direction; and (c) relative energies along the [11̅0] direction. The Tkatchenko–Scheffler (TS) dispersion correction was used with the PBE, HSE03, and HSE06 density functional approximations (DFAs). No dispersion correction was applied with the revTPSS DFA.

Figure 8 shows the relative energies of a single gold atom along the [001] and [11̅0] directions with respect to the initial adsorption site. The curves are not symmetrical around the origin, as the relaxed structure is asymmetrical along the [001] and [11̅0] axes close to the defect. In general, the surfaces that lead to stronger adsorption of the single gold atom have larger energetic barriers along both directions. More specifically, the introduction of defects and dopants increases the stability of the single gold atom with greater kinetic barriers observed. The result occurs because the gold adsorbate is more strongly bound to these surfaces, which means more energy would be required to overcome the interaction and translate the gold atom across the diamond surface.

As shown in Figure 8b,c, the barriers to leaving the adsorption site on the pristine surface are quite low compared to those on defective and doped surfaces. For the pristine surface, a low barrier is observed because the gold atom is not strongly bound to the surface, as shown in Table 1 and Figure 5. The energetic barriers to move the gold atom along the [001] direction were calculated to be 0.14 and 0.18 eV for the negative and positive displacements, respectively, with PBE^+TS^/REBO, which are in close agreement with the energy barrier of 0.16 eV that was predicted by Hussein et al. using a periodic PBE^+TS^-optimized model of the pristine surface.^36^ The relative energies along the [11̅0] direction were generally higher, with barriers using the same method of 0.24 and 0.21 eV for the negative and positive displacements, respectively, which are comparable to the energy barrier of 0.25 eV predicted by Hussein et al. using a periodic surface model.^36^ The higher barriers along the [11̅0] direction relative to the [001] direction are expected, as the gold atom has to move above the plane of carbonyl oxygen atoms that lie along this axis, as shown by the purple arrows in Figure 8a. In contrast, along the [001] direction, the gold atom moves above the plane of ether oxygen atoms to move across the surface (blue arrows in Figure 8a). The ether oxygen atoms are located at a lower height than the carbonyl oxygen atoms, with respect to the surface carbon atoms. The gold adatom, therefore, can translate at a lower height along the [001] direction, as opposed to the [11̅0] direction, resulting in a lower energy barrier.

While the PBE^89^ GGA was shown to perform well with respect to other DFAs for the prediction of adsorption energetics on the idealized system above, the embedded cluster approach facilitates a further comparison of barriers using the HSE03^135^ and HSE06^136^ HGGAs. Both of these HGGAs predict similar relative energies; in Figure 8b, for the [001] direction, the HGGA barriers are similar to that calculated for PBE: 0.12 and 0.15 eV along the negative and positive displacements, respectively. There is some difference in the shapes of their curves along the [11̅0] direction, compared to PBE; however, HSE03 and HSE06 predict energy barriers of 0.15 and 0.17 eV, respectively, for the positive displacement, and a barrier of 0.23 eV for the negative displacement, which are only slightly lower than the PBE value.

Unlike the pristine surface, the SCOV-defective surface displays large barriers to diffusion. The large barriers are expected, as the gold atom is chemisorbed at the defect. For the [001] direction, as shown in Figure 8b, the barriers are calculated to be 1.93 and 6.86 eV along the negative and positive displacements, respectively, using PBE^+TS^/REBO. The disparity between the displacement can be explained by the structural asymmetry; along the negative displacement, shown in Figure 3b, the surface is hydrogen-terminated in the neighborhood of the gold atom, which means a lower energy would be required to move the atom across the surface than along the positive displacement, where the gold atom has to move above a carbonyl oxygen atom. For the [11̅0] direction, the predicted energy barriers are also very high, at 2.28 and 1.96 eV along the negative and positive displacements, respectively, using PBE^+TS^/REBO. The accuracy of PBE was benchmarked against the revTPSS^134^ MGGA, which was shown to perform similarly to PBE for the SCOV-defective surface. The calculated curves and energy barriers with revTPSS agree very well with PBE, as can be seen in Figure 8b,c.

Substituting a carbon atom with an explicit boron dopant in the surface layers of the diamond substrate also increases the kinetic stability of the gold atom compared to that of the pristine surface. For the [001] direction, the barrier along the negative displacement is larger when the boron dopant is in the third layer (0.74 eV) than in the second layer (0.16 eV). However, the barrier along the positive displacement is larger when the boron dopant is in the second layer (0.62 eV) rather than in the third layer (0.45 eV). Along the [11̅0] direction, the boron dopant in the third layer results in a barrier of 1.03 eV along the negative displacement, whereas the second-layer boron results in a lower barrier of 0.39 eV. Along the positive displacement, the second- and third-layer barriers are 0.89 and 1.04 eV, respectively. These barriers are lower than for the SCOV-defective surface but clearly show an increase in stability for the single gold atom compared to the idealized pristine surface.

In general, adsorption on the pristine fully oxygenated diamond (110) surface results in low kinetic barriers for the single gold atom, but the introduction of defects or dopants into the surface significantly increases the adsorption energy of the gold atom when adsorbed directly on these defects. Similar to the trend observed with adsorption energies, the barriers associated with explicitly modeled boron dopants were not as large as those associated with the SCOV defect, though both increase the stability of the single gold atom on the surface. Furthermore, the barriers predicted for the idealized and SCOV-defective surfaces were robust with respect to a range of DFAs. The barriers calculated for the defect sites suggest that thermally activated diffusion of the gold atom during baking before transfer to the microscope should be rare. The low barriers associated with the pristine surface, on the other hand, are unlikely to prevent diffusion during thermal baking or as induced by the high-energy electron beam in electron microscopy experiments. The high stability of single gold atoms on BDD observed by Hussein et al.^36^ during STEM measurements is only consistent with strong adsorption in defect sites. The finding has interesting implications for metal nanocluster nucleation, as it suggests that single metal atoms are preferably formed at surface defect sites (either vacancies or charged defects) on BDD. Once formed, the nucleation sites are highly stable and will seed further growth. Interestingly, Hussein et al. saw few instances of dimers or few-atom clusters, which might indicate that small clusters might be removed in the *ex situ* sample preparation, leaving only the defect-stabilized single atom behind.

## Conclusions

Embedded QM/MM cluster models have been used to study the adsorption energetics of single gold atoms on oxygen-terminated diamond (110) surfaces as well as to analyze the effects of local surface defects and dopants on adsorption energies. For the pristine, fully oxygenated surface, the gold atom weakly adsorbs onto the surface. The introduction of defects and boron dopants into the surface substrate, however, significantly increases the adsorption energy of a single gold atom. In the former case, the introduction of a SCOV into the surface results in strong adsorption of the gold adatom, and the interaction between the adatom and a surface ether oxygen atom was found to have attributes of a polar covalent bond and an ionic bond.

After the identification of stabilization mechanisms for the single gold atom, the validity of the trends observed using the PBE^+TS^/REBO method was evaluated by benchmarking the method against other force fields, dispersion correction schemes, and DFAs. The REBO force field was shown to be an appropriate embedding environment for the QM region, while little dependency was found on the flavor of dispersion correction, though a dispersion correction is necessary to accurately capture the adsorption energetics of the single gold adatom at the GGA level. The PBE GGA generally performs very well with respect to other GGAs, as well as higher-rung MGGAs and HGGAs, for calculating adsorption energies. We conclude that the dispersion-corrected PBE GGA remains an appropriate choice to treat the physisorbed and chemisorbed interactions. Some disagreement was identified between (M)GGAs and higher-rung HGGAs for the delocalized triel-doped surface, which is because the lower-rung DFAs fail to fully capture the mid- to long-range interactions, and HSE06 or PBE0 might be more appropriate DFA choices to treat the charged defect. However, all DFAs predicted stronger adsorption of the single gold atom on defective and doped surfaces compared to the pristine surface, indicating that the observed relative trends in adsorption are robust with respect to the choice of DFA.

Finally, the embedded cluster models were used to investigate the thermal stability of single gold atoms in their adsorption sites and to analyze the effects of local surface defects on diffusion. The diffusion barriers associated with the pristine surface along both the [001] and [11̅0] directions are very low, and as a result, the pristine surface is unlikely to stabilize single gold atoms when studied under experimental conditions. The introduction of defects and boron dopants into the surface substrate, however, significantly increases the energetic barriers associated with the lateral diffusion of the gold adatom along the surface.

The results outlined herein indicate that the high stability of single gold atoms on polycrystalline BDD surfaces observed by Hussein et al.^36^ is most likely due to surface defects and dopants that are not observable in STEM images or accounted for within previous calculations. Furthermore, this work shows that the first step of metal deposition, namely, the adsorption of a single metal atom, will likely occur at surface defect sites, but the details of further growth of clusters from adatoms remain unclear. There are only few instances in which compact clusters below 10 atoms are seen in the STEM images of Hussein et al., which suggests that the critical size for clusters to withstand thermal baking may be larger. The exact atomistic thermodynamics and kinetics of nanocluster growth need further investigation. This work forms the foundation for wider efforts to model single-atom and nanocluster deposition and the properties of hybrid metal/carbon-based interfaces, and showcases how these can be facilitated by embedded cluster and QM/MM approaches.

## Data Availability

Input and output files for all calculations have been uploaded as a data set to the NOMAD electronic structure data repository and are freely available under 10.17172/NOMAD/2023.04.19-1. A preprint of this paper is available on arXiv.^162^

## Supporting Information Available

Further details on QM region size optimization, computational scaling of QM/MM versus periodic QM, benchmarking of force fields and dispersion correction schemes, conformational isomers of SCOV-defective surfaces, and binding energy curves of SCOV-defective surfaces (PDF)
