# Supplementary material for: Stability of Single Gold Atoms on Defective and Doped Diamond Surfaces
Source: J Phys Chem C Nanomater Interfaces. 2023 Aug 7;127(32):16187–203. doi: 10.1021/acs.jpcc.3c03900 (PMC10440818; doi:10.1021/acs.jpcc.3c03900)
Supplement: Supplementary file 1 — jp3c03900_si_001.pdf [file jp3c03900_si_001.pdf]

Supporting Information: ‘Stability of Single Gold Atoms on Defective and Doped Diamond Surfaces’

Shayantan Chaudhuri,[†,‡] Andrew J. Logsdail,[¶] and Reinhard J. Maurer[*,†,§]

†*Department of Chemistry, University of Warwick, Coventry, CV4 7AL, United Kingdom*

‡*Centre for Doctoral Training in Diamond Science and Technology, University of Warwick, CV4 7AL, United Kingdom*

¶*Cardiff Catalysis Institute, School of Chemistry, Cardiff University, Cardiff, CF10 3AT, United Kingdom*

§*Department of Physics, University of Warwick, Coventry, CV4 7AL, United Kingdom*

E-mail: r.maurer@warwick.ac.uk

Contents

1 QM Region Size Optimization **S-3**

2 Scaling of QM/MM versus Periodic QM **S-6**

3 Benchmarking MM Forcefields **S-7**

4 Benchmarking Dispersion Correction Schemes **S-8**

5 Conformational Isomers of SCOV-Defective Surfaces **S-13**

6 Binding Energy Curves for SCOV-Defective Surfaces **S-16**

7 Benchmarking Density-Functional Approximations **S-17**

References **S-18**

1 QM Region Size Optimization

It is important to ensure that the size of the QM region embedded within the MM region is large enough to avoid any finite-size effects. The appropriate QM region size of the substrate was chosen by comparing various properties of PBE^{+TS}/REBO-optimized embedded cluster models with varying QM region size against the parent PBE^{+TS}-optimized periodic model. All convergence tests were conducted on an idealized surface model. First, the structural deviations of each PBE^{+TS}/REBO-optimized embedded cluster were compared against the initially cut cluster from the PBE^{+TS}-optimized periodic model, which can therefore be taken to be an appropriate representation of the periodic model. As can be seen from Figure S1(a), PBE^{+TS}/REBO-optimized embedded clusters with QM region sizes of 10, 20, 60, 70 and 90 atoms have the lowest root-mean-square deviation (RMSD), 0.037 Å, with respect to the PBE^{+TS}-optimized periodic model, while the RMSDs for the 30-, 40-, 50- and 80-atom QM regions were at least 0.7 eV, showing a greater disparity in optimized structures.

Following the structural comparison, the electronic structures of the PBE^{+TS}/REBO-optimized embedded cluster models were compared against the PBE^{+TS} periodic model. Figure S1(b) shows a graph of the band gap, which is the energy between the highest occupied molecular orbital and the lowest unoccupied molecular orbital, of the embedded clusters as a function of QM region size. The band gap generally decreases as the QM region size increases and tends towards the QM periodic value (2.3 eV). This shows that clusters with smaller QM region sizes do experience some finite-size effects. In contrast, as the QM region size increases, the embedded cluster becomes more structurally and energetically similar to the periodic QM structure. Despite this overall trend, over the range of QM region sizes explored, the 30-atom QM region was found to possess the closest band gap to the periodic value, while the 90-atom QM region had the next closest value (2.6 eV). The band gaps of the 10-, 20-, 60- and 70-atom QM regions, which were found to be structurally similar to the periodic model, were found to be at least 0.5 eV larger than the QM periodic value.

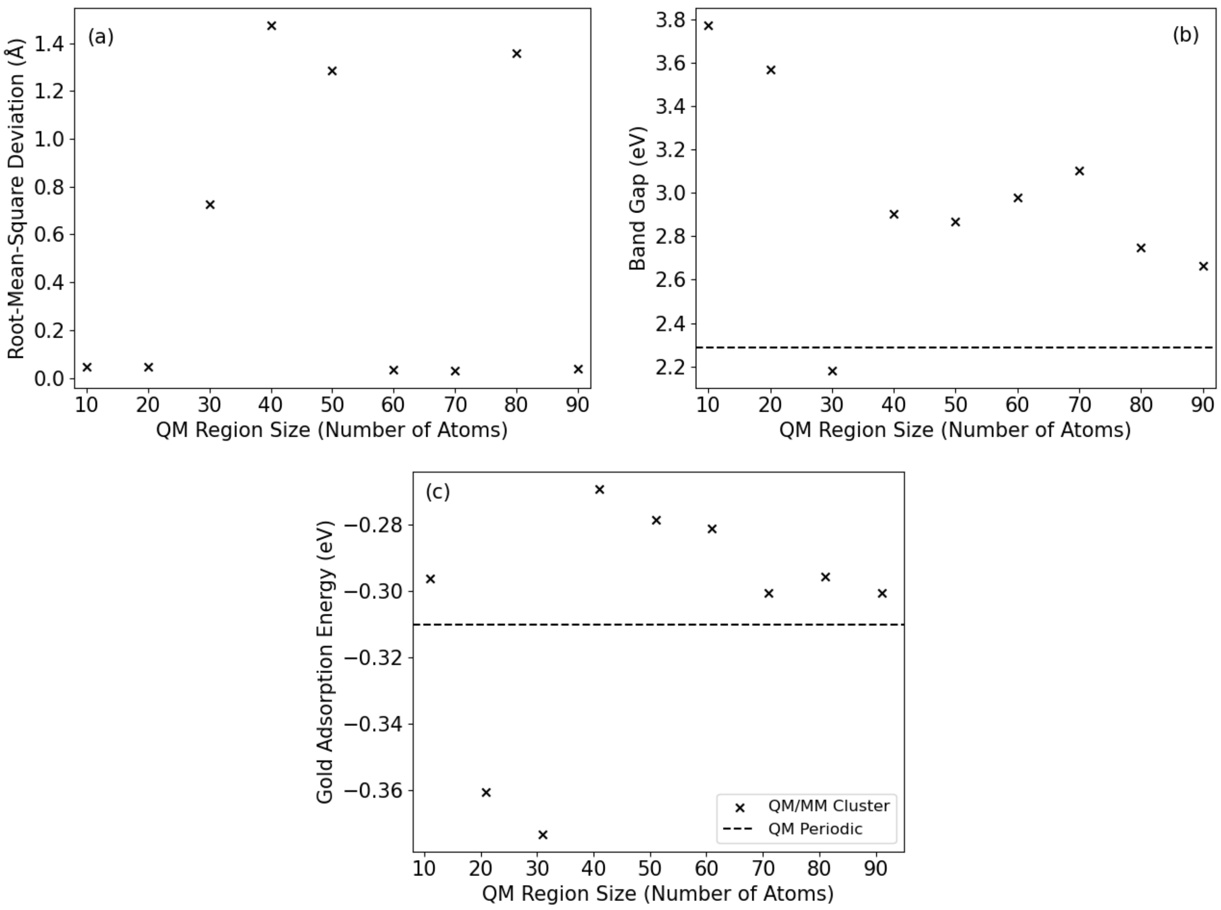

Figure S1. Scatter graphs showing the (a) root-mean-square deviations and (b) band gaps of a single gold atom atop PBE^{+TS}/REBO-optimized cluster models against the initial cluster cut from the PBE^{+TS}-optimized periodic model; and (c) adsorption energies of a single gold atom atop PBE^{+TS}/REBO-optimized cluster models against the PBE^{+TS}-optimized periodic model, all as a function of QM region size.

Finally, the adsorption energy of a single gold atom was evaluated as a function of the QM region size, as shown in Figure S1(c). The 70- and 90-atom QM regions (71- and 91-atoms respectively including the gold atom) resulted in the closest adsorption energy ($-0.30\,\mathrm{eV}$) to the QM periodic value ($-0.31\,\mathrm{eV}$). The 20- and 30-atom QM regions significantly overestimate the adsorption energy, while the 10-, 40-, 50-, 60- and 80-atom QM regions report similar adsorption energetics to the QM periodic model, but are not as close as the 70- and 90-atom QM regions.

Taking all results into consideration, the 90-atom QM region results in an optimized structure, band gap and gold adsorption energy most similar to the QM periodic model, and was thus chosen as the optimal QM region size within the QM/MM cluster. While a larger QM region would most likely result in a cluster with a final geometry and energetics more similar to the periodic model, convergence problems were encountered with larger QM region sizes (100 and 110 atoms). Regardless, the embedded cluster with a 90-atom QM region was found to have similar structural and energetic properties to the periodic QM cluster and was thus deemed an appropriate size.

2 Scaling of QM/MM versus Periodic QM

To showcase the higher computational efficiency of the hybrid QM/MM approach, scaling graphs were constructed after conducting single-point calculations on the PBE^{+TS}-optimized periodic model and the PBE^{+TS}/REBO-optimized model, with a 90-atom QM region, after a gold atom was adsorbed onto the model surfaces. Figure S2 shows the computational cost of these single-point calculations as a function of number of cores, which were run on Lenovo NeXtScale nx360 M5 servers with dual Intel Xeon E5-2680 v4 (Broadwell) 14-core processors at 2.4 GHz, as available within the Orac high performance computing cluster provided by the Scientific Computing Research Technology Platform of the University of Warwick. All calculations used the Eigenvalue SoLvers for Petaflop-Applications[S1] library and the ELectronic Structure Infrastructure.[S2]

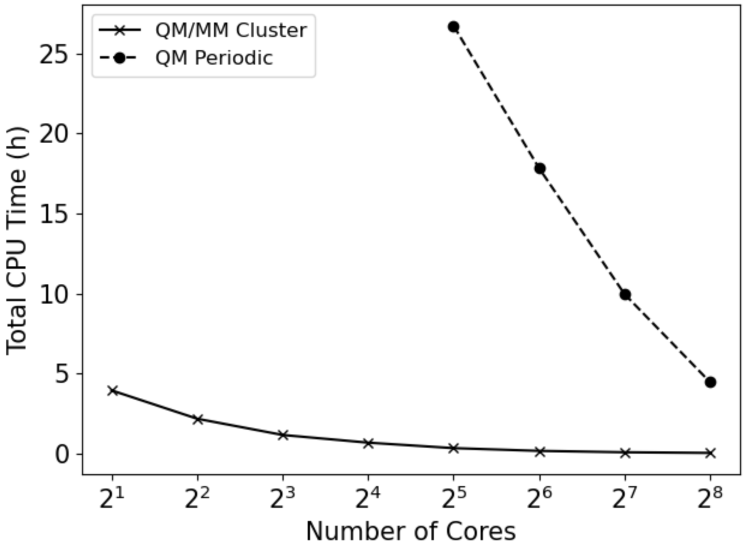

Figure S2. Scaling graphs of single-point PBE^{+TS}/REBO and PBE^{+TS} calculations of the idealized oxygen-terminated diamond (110) surface. The embedded cluster model comprised 527 atoms with a 90-atom QM region, while the periodic model comprised a 92-atom unit cell.

As can be seen in Figure S2, QM/MM calculations are vastly cheaper than the periodic QM calculations. Furthermore, periodic QM calculations failed when using 16 cores or fewer due to memory issues. This confirms the superior computational efficiency of the hybrid

QM/MM approach and that it can be used to access more computationally-costly methods such as MGGAs and HGGAs.

3 Benchmarking MM Forcefields

It is important to ensure that the adsorption energetics of a single gold atom are not significantly affected by the choice of the embedding forcefield environment. To investigate further, the adsorption energy of a single gold atom on an idealized surface, as calculated using PBE^{+TS}/REBO, was benchmarked against the Tersoff[S3] forcefield, where PBE^{+TS} was used as the complementary QM method. Similar to REBO, the Tersoff forcefield was developed specifically for carbon, with applications to amorphous carbon,[S3] and is thus an appropriate forcefield to benchmark the REBO forcefield against.

Table S1 benchmarks the REBO forcefield against the Tersoff forcefield. Both the REBO and Tersoff forcefields result in the same adsorption energy for a single gold atom on an idealized surface. Furthermore, there is a very small disparity in adsorption height (0.05 Å), showing that both forcefield methods predict virtually identical adsorption energetics for the single gold atom, and that the REBO forcefield is appropriate to embed the QM region within.

Table S1. Adsorption energetics and heights of a single gold atom adsorbed onto an idealized oxygen-terminated diamond (110) surface after a PBE^{+TS}/MM optimization, using various MM forcefield methods.

MM Forcefield	Adsorption Height (Å)	Adsorption Energy (eV)
REBO[S4,S5]	1.71	−0.30
Tersoff[S3]	1.76	−0.30

4 Benchmarking Dispersion Correction Schemes

It is important to ensure long-range dispersion effects such as van der Waals (vdW) forces are treated appropriately, as they can have a significant effect on the adsorption structure. The pairwise additive TS scheme does not explicitly account for beyond-pairwise vdW interactions. For this reason, the TS scheme was benchmarked against some *a posteriori* many-body dispersion (MBD)[S6,S7] correction schemes, namely the range-separated self-consistently screened (MBD@rsSCS)[S8] and the non-local (MBD-NL)[S9] variants. PBE^{+TS}/REBO was also benchmarked against non-dispersion-corrected PBE i.e. PBE/REBO calculations. The performance of various dispersion corrections was benchmarked by comparing the final adsorption energy and adsorption height after a full dispersion-corrected PBE/REBO geometry optimization, and by constructing binding energy curves by running a series of dispersion-corrected PBE/REBO single-point calculations with the gold adatom placed at various heights above the surface, which represent the variation of the adsorption energy as a function of adsorption height.

Dispersion corrections were benchmarked on the idealized, SCOV-defective, and delocalized triel-doped systems. The idealized and SCOV-defective systems were chosen as they would permit dispersion corrections to be benchmarked on both 'more physisorbed' and 'more chemisorbed' systems, respectively. The delocalized triel-doped system was chosen over the localized boron-doped models for three reasons: firstly, with common boron dopant densities, the probability of the dopant atom being within the bulk material is much higher than it being in the top surface layers. Secondly, the delocalized model is applicable to any triel dopant and thirdly, the predicted adsorption height and energy do not differ significantly from the localized case with the boron dopant in the third layer, as shown in the main text.

Table S2 details the adsorption heights and energies of a single gold atom after TS-,

Table S2. Adsorption energetics and heights of a single gold atom adsorbed onto various oxygen-terminated diamond (110) surfaces after a dispersion-corrected PBE/REBO optimization, using various *a posteriori* dispersion correction schemes. No data were attained using MBD@rsSCS for the SCOV-defective system due to the negative polarizabilities for some atoms after the initial FHI-aims calculation settings.

Dispersion Correction	Adsorption Height (Å)	Adsorption Energy (eV)
Idealized Surface		
TS[S10]	1.71	−0.30
MBD@rsSCS[S8]	1.60	−0.29
MBD-NL[S9]	1.63	−0.27
No Dispersion	1.82	−0.15
SCOV-Defective Surface		
TS	−0.17	−2.31
MBD@rsSCS	–	–
MBD-NL	−0.13	−2.29
No Dispersion	−0.11	−2.04
Delocalized Triel-Doped Surface		
TS	0.36	−1.97
MBD@rsSCS	1.06	−1.73
MBD-NL	1.06	−1.71
No Dispersion	1.09	−1.53

MBD@rsSCS-, MBD-NL-, and non-dispersion-corrected PBE/REBO optimizations of the idealized, SCOV-defective, and delocalized triel-doped surfaces. For the idealized surface, there is very little disparity between TS and the MBD approaches with respect to both adsorption heights and energies. Both MBD@rsSCS and MBD-NL perform very similarly to each other, and predict slightly weaker adsorption than TS despite the gold atom adsorbing closer to the surface; however, these differences are minor (0.11 Å and 0.03 eV at most for adsorption heights and energies, respectively). The lack of a dispersion correction does have a more evident effect, with the gold atom adsorbing 0.11 Å higher than with TS and 0.22 Å higher than with MBD@rsSCS. Furthermore, the adsorption energy of the gold atom was even weaker without a dispersion correction. The non-dispersion-corrected results are in

line with literature, where non-dispersion-corrected PBE, as well as other GGAs, have been observed to underestimate adsorption energies and overestimate adsorption distances,[S11–S14] and highlights the importance of including a dispersion correction with such DFAs.

For the SCOV-defective surface, the TS method once again performs quite well with respect to MBD-NL. After a PBE$^{+\text{MBD-NL}}$/REBO optimization, the gold atom adsorbs 0.04 Å higher than with TS, and this small disparity is reflected in the adsorption energy, which is only 0.02 eV weaker than with TS. No data were attained using MBD@rsSCS for this system, due to the negative polarizabilities for some atoms after the initial FHI-aims calculation settings, which prevented the MBD@rsSCS calculation from completing. It should be noted that this is a technical limitation of the MBD@rsSCS approach that can occur in some systems under certain conditions, and is not physically meaningful. Without any dispersion correction, the gold atom again adsorbs higher than dispersion-corrected approaches, and the adsorption energy was evaluated to be at least 0.25 eV weaker, further attesting to the need for a dispersion correction.

Finally, for the delocalized triel-doped system, there does appear to be some dependency on the choice of dispersion correction. The TS correction predicts stronger adsorption than both MBD approaches, and the gold atom optimizes to a site far closer to the surface. The MBD approaches perform very similar to each other, with the gold atom predicted to adsorb 1.06 Å above the surface with an adsorption energy just larger than -1.7 eV. The consistency between the MBD approaches and the relatively weaker adsorption energies are indicative of the beyond-pairwise interactions being taken into account, and these effects have a greater influence within this charged system rather than the neutral idealized and SCOV-defective systems. The lack of a dispersion correction yet again resulted in the gold atom adsorbing higher than dispersion-corrected approaches, and the adsorption energy was evaluated to be at least 0.18 eV weaker.

Figure S3 shows the binding energy curves using various dispersion-corrected PBE/REBO calculations on the idealized, SCOV-defective, and delocalized triel-doped surfaces. Figure S3(a) shows the curves for the idealized surface, where all dispersion-corrected curves have similar shapes, with adsorption energy minima between −0.12 eV and −0.11 eV at an adsorption height of 3.0 Å. The results show that there is no major dependency on which dispersion correction is used. The MBD approaches predict only slightly weaker adsorption than the pairwise TS scheme. However, without any dispersion correction, the adsorption energy minimum reduces to −0.05 eV, indicating very little, near-zero adsorption, as has been reported in Table S2 and in literature.[S11–S14]

Figure S3(b) shows the binding energy curves for the SCOV-defective surface, where all dispersion-corrected PBE/REBO[S15] curves have similar shapes, with adsorption energy minima between −0.27 eV and −0.19 eV at an adsorption height of 3.5 Å with TS and MBD-NL, respectively. The results show that there is a slight disparity depending on what dispersion correction is used, but it is not a major difference. Furthermore, the minima of the curves are much deeper for the SCOV-defective surface than the idealized surface. Without any dispersion correction, the adsorption energy minimum reduces to −0.05 eV, which is very similar to that of the idealized surface, and further showcases the importance of including a dispersion correction for the SCOV defect.

Finally, all the curves for the delocalized triel-doped surface have a minimum at a height of 2.0 Å, as can be seen in Figure S3(c). For this surface, both TS and MBD@rsSCS have near-identical results, with an adsorption energy value of −0.76 eV at 2.0 Å. MBD-NL predicts a similar curve to these two dispersion schemes, with an adsorption energy value of −0.73 eV at 2.0 Å, though some differences arise at around 3.0 Å. Yet again, a lack of a dispersion correction results in a shallower curve with an adsorption energy of −0.60 eV at 2.0 Å.

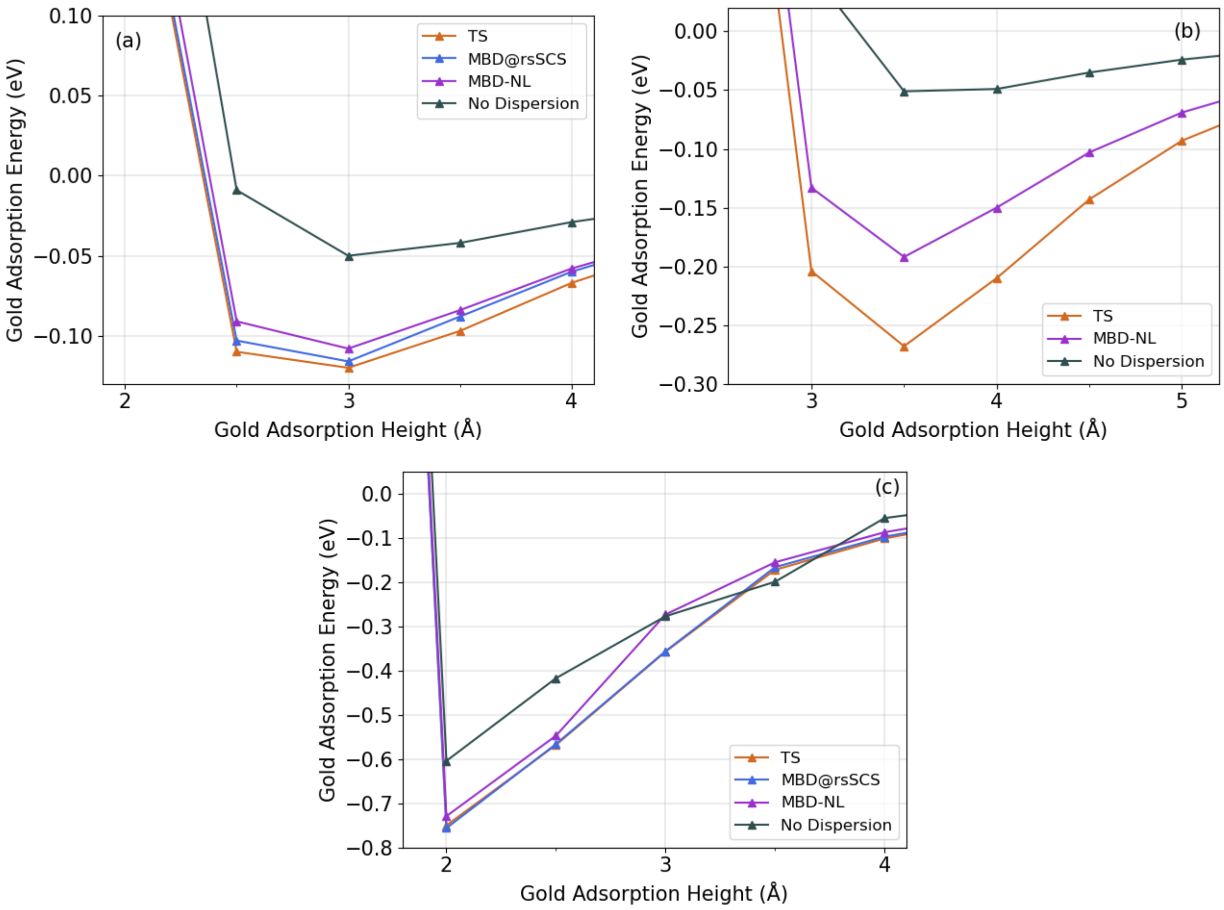

Figure S3. Binding energy curves showing the adsorption energy of a single gold adatom on various oxygen-terminated diamond (110) surface substrates as a function of height above the plane of carbonyl oxygen atoms on the substrate surface after dispersion-corrected PBE/REBO calculations. Substrates are (a) an idealized oxygen-terminated diamond (110) surface (b) a SCOV-defective surface and (c) a delocalized triel-doped surface.

Overall, after comparing the TS scheme against the MBD@rsSCS and MBD-NL dispersion schemes, no major dependency on the flavor of dispersion correction can be observed. However, a lack of a dispersion correction results in a weaker adsorption energy and a larger adsorption height, showing the importance of accounting for van der Waals effects if the DFA does not include any mid-/long-range dispersion interactions.

5 Conformational Isomers of SCOV-Defective Surfaces

To ensure the SCOV defect was correctly modeled with every DFA, DFA_i, the carbonyl oxygen was first removed and the surface was reoptimized using DFA_i/REBO. After this initial optimization, the surface structure at the defect site, centred at the former carbonyl carbon atom, should change from bent (originally trigonal planar with the carbonyl oxygen atom in the idealized system) to trigonal pyramidal. Because diamond surfaces are usually hydrogen-terminated after chemical vapor deposition growth,[S16] uncoordinated carbon atoms were subsequently saturated with hydrogen species and the surface was reoptimized using DFA_i/REBO, after which the surface structure at the defect site should change to tetrahedral. Based on valence shell electron pair repulsion theory, this shows a return of the •C• atom to an sp^3-hybridized state. This is the correct surface configuration as an oxygen atom is needed to pull the carbon atom above the diamond (110) surface plane to form a carbonyl group at the surface.[S17] Without this oxygen atom, the carbon atom would remain in an sp^3-hybridized configuration.

Only the DFAs that returned the former-carbonyl carbon atom to an sp^3-hybridized configuration were investigated further. This was evaluated by studying the conformational isomerism of the structure centered at the former-carbonyl carbon atom. After the removal of the carbonyl oxygen atom and optimization with a given DFA, the dihedral angle between a surface ether oxygen atom and a surface carbon atom, along the bond between the former-carbonyl and corresponding ether carbon atoms, was calculated. Table S3 details the calculated dihedral angles after optimization with various DFAs. Most DFAs correctly return the structure to a synclinal conformation, with dihedral angles of approximately 60°, which indicates a more sp^3-hybridized configuration. However, all investigated HGGAs result in an anticlinal conformation, with dihedral angles of approximately 150°, which indicates the former-carbonyl carbon atom remains in a more sp^2-hybridized state. Even though the higher-rung HGGAs predict the anticlinal conformation, this is most likely a local energy

minimum and as explained above, is not the correct physical conformation for the surface after the removal of a carbonyl oxygen atom. In order to ensure lower-rung DFAs could still be benchmarked against HGGAs, the final PBE^{+TS}/REBO-optimized SCOV-defective structures were reoptimized using the respective HGGA^{+TS}/REBO method. Table S4 details the difference between the energy of the synclinal conformation and the energy of the anticlinal conformation, as calculated using various TS-corrected HGGAs. As can be seen in Table S4, the energy difference between the two conformations is between 0.73–0.86 eV, indicating the greater stability of the synclinal conformation. For clarity, the Newman projections[S18] of the synclinal and anticlinal conformations are also provided in Figure S4.

Table S3. Dihedral angles between a surface ether oxygen atom and a surface carbon atom, along the bond between the former-carbonyl and corresponding ether carbon atoms after the removal of a carbonyl oxygen atom from the idealized oxygen-terminated diamond (110) surface and subsequent optimization with various DFAs. DFAs are ordered from low- to high-rung, along Jacob's ladder,[S32] and the TS[S10] dispersion correction method was applied to all GGAs and HGGAs.

DFA	Dihedral Angle (°)
PZ-LDA[S19,S20]	54.3
KSDT[S21]	54.3
PBE[S15]	55.7
revPBE[S22]	55.2
RPBE[S23]	56.1
PBEsol[S24]	55.1
TPSS[S25]	56.4
TPSSloc[S26]	56.0
revTPSS[S27]	56.3
PBE0[S28]	141.3
PBEsol0[S29]	140.9
HSE03[S30]	141.1
HSE06[S31]	141.1

Table S4. Relative energies between the synclinal and anticlinal conformations of the SCOV-defective substrate surface, as calculated using TS-corrected HGGAs.

HGGA	Relative Energy (eV)
PBE0[S28]	−0.73
PBEsol0[S29]	−0.86
HSE03[S30]	−0.75
HSE06[S31]	−0.74

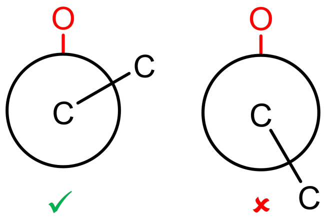

Figure S4. Newman projections[S18] of the synclinal (left) and anticlinal (right) conformations after optimization with various DFAs. The projection is along a bond between the former-carbonyl carbon atom and a surface ether carbon atom. The synclinal conformation is the correct model for the SCOV defect (prior to hydrogen saturation).

6 Binding Energy Curves for SCOV-Defective Surfaces

Figure S5 shows the binding energy curves for the SCOV-defective surface, as calculated with various DFAs. As can be seen, LDAs and most GGAs predict similar binding energy curves. The revPBE and PZ-LDA DFAs predict the strongest adsorption at an adsorption height of 3.0 Å. PBE and PBEsol have similar curves to each other. The RPBE GGA predicts the weakest adsorption among GGAs and has a binding energy minimum at 4.0 Å, which is a larger adsorption height value than all other LDAs and GGAs, much like in the idealized case. In contrast, all MGGAs result in very shallow binding energy curves for the single adatom. Furthermore, the binding energy curve for TPSS, much like RPBE, has a minimum at a value larger than all other DFAs. However, the revTPSS binding energy curve is very similar to other MGGAs' despite the strong adsorption predicted in Figure 6. Much like with the MGGAs, the PBEsol0 HGGA also has a shallow binding energy curve, while the other HGGA binding energy curves are very similar to the PBE binding energy curve.

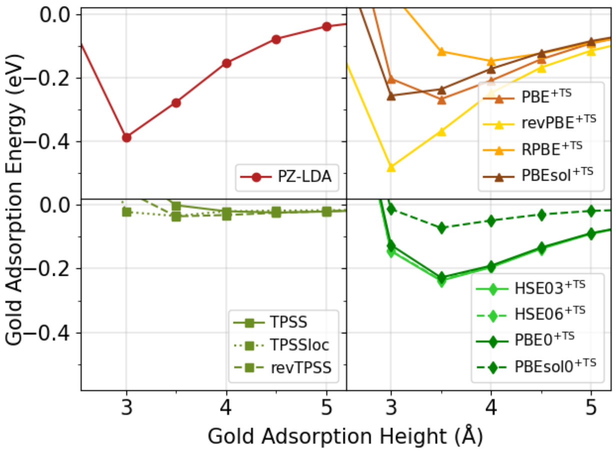

Figure S5. Unrelaxed binding energy curves showing the adsorption energy of a single gold adatom as a function of height above an oxygen-terminated diamond (110) surface with a saturated carbonyl oxygen vacancy defect. Density-functional approximations are divided according to (from left to right): local-density approximations (LDAs), Tkatchenko-Scheffler (TS)-corrected generalized gradient approximations (GGAs), meta-GGAs (MGGAs), and TS-corrected hybrid GGAs (HGGAs).

7 Benchmarking Density-Functional Approximations

Table S5. Adsorption energies for a single gold adatom on various oxygen-terminated diamond (110) surface substrates. Adsorption heights are given with respect to the plane of carbonyl oxygen atoms. The TS dispersion correction was used alongside the PBE, PBE0 and HSE06 DFAs, but not with revTPSS.

System	Adsorption Energy (eV)			
	PBE	revTPSS	PBE0	HSE06
Idealized	−0.30	−0.15	−0.23	−0.24
SCOV-defective	−2.31	−2.20	−2.22	−2.24
Delocalized triel-doped	−1.98	−2.21	−2.70	−2.50

Table S6. Adsorption heights for a single gold adatom on various oxygen-terminated diamond (110) surface substrates. Adsorption heights are given with respect to the plane of carbonyl oxygen atoms. The TS dispersion correction was used alongside the PBE, PBE0 and HSE06 DFAs, but not with revTPSS.

System	Adsorption Height (Å)			
	PBE	revTPSS	PBE0	HSE06
Idealized	1.71	1.62	1.62	1.70
SCOV-defective	−0.12	−0.16	−0.17	−0.16
Delocalized triel-doped	0.36	0.31	1.07	1.07

References

(S1) Marek, A.; Blum, V.; Johanni, R.; Havu, V.; Lang, B.; Auckenthaler, T.; Heinecke, A.; Bungartz, H.; Lederer, H. The ELPA library: scalable parallel eigenvalue solutions for electronic structure theory and computational science. *J. Phys. Condens. Matter* **2014**, *26*, 213201.

(S2) Yu, V. W.-z.; Corsetti, F.; García, A.; Huhn, W. P.; Jacquelin, M.; Jia, W.; Lange, B.; Lin, L.; Lu, J.; Mi, W., et al. ELSI: A unified software interface for Kohn–Sham electronic structure solvers. *Comput. Phys. Commun.* **2018**, *222*, 267–285.

(S3) Tersoff, J. Empirical Interatomic Potential for Carbon, with Applications to Amorphous Carbon. *Phys. Rev. Lett.* **1988**, *61*, 2879–2882.

(S4) Brenner, W. D.; Shenderova, O. A.; Harrison, J. A.; Stuart, S. J.; Ni, B.; Sinnott, S. B. A second-generation reactive empirical bond order (REBO) potential energy expression for hydrocarbons. *J. Phys. Condens. Matter* **2002**, *14*, 783–802.

(S5) Ni, B.; Lee, K.-H.; Sinnott, S. B. A reactive empirical bond order (REBO) potential for hydrocarbon–oxygen interactions. *J. Phys. Condens. Matter* **2004**, *16*, 7261–7275.

(S6) Tkatchenko, A.; DiStasio Jr., R. A.; Car, R.; Scheffler, M. Accurate and Efficient Method for Many-Body van der Waals Interactions. *Phys. Rev. Lett.* **2012**, *108*, 236402.

(S7) Tkatchenko, A.; Ambrosetti, A.; DiStasio Jr., R. A. Interatomic methods for the dispersion energy derived from the adiabatic connection fluctuation-dissipation theorem. *J. Chem. Phys.* **2013**, *138*, 074106.

(S8) Ambrosetti, A.; Reilly, A. M.; DiStasio Jr., R. A.; Tkatchenko, A. Long-range correlation energy calculated from coupled atomic response functions. *J. Chem. Phys.* **2014**, *140*, 18A508.

(S9) Hermann, J.; Tkatchenko, A. Density Functional Model for van der Waals Interactions: Unifying Many-Body Atomic Approaches with Nonlocal Functionals. *Phys. Rev. Lett.* **2020**, *124*, 146401.

(S10) Tkatchenko, A.; Scheffler, M. Accurate Molecular Van Der Waals Interactions from Ground-State Electron Density and Free-Atom Reference Data. *Phys. Rev. Lett.* **2009**, *102*, 073005.

(S11) Maurer, R. J.; Freysoldt, C.; Reilly, A. M.; Brandenburg, J. G.; Hofmann, O.; Björkman, T.; Lebègue, S.; Tkatchenko, A. Advances in Density-Functional Calculations for Materials Modeling. *Annu. Rev. Mater. Sci.* **2019**, *49*, 1–30.

(S12) Hofmann, O. T.; Zojer, E.; Hörmann, L.; Jeindl, A.; Maurer, R. J. First-principles calculations of hybrid inorganic–organic interfaces: from state-of-the-art to best practice. *Phys. Chem. Chem. Phys.* **2021**, *23*, 8132–8180.

(S13) Ruiz, V. G.; Liu, W.; Zojer, E.; Scheffler, M.; Tkatchenko, A. Density-Functional Theory with Screened van der Waals Interactions for the Modeling of Hybrid Inorganic-Organic Systems. *Phys. Rev. Lett.* **2012**, *108*, 146103.

(S14) Maurer, R. J.; Ruiz, V. G.; Camarillo-Cisneros, J.; Liu, W.; Ferri, N.; Reuter, K.; Tkatchenko, A. Adsorption structures and energetics of molecules on metal surfaces: Bridging experiment and theory. *Prog. Surf. Sci.* **2016**, *91*, 72–100.

(S15) Perdew, J. P.; Burke, K.; Ernzerhof, M. Generalized Gradient Approximation Made Simple. *Phys. Rev. Lett.* **1996**, *77*, 3865–3868.

(S16) Balmer, R. S.; Brandon, J. R.; Clewes, S. L.; Dhillon, H. K.; Dodson, J. M.; Friel, I.; Inglis, P. N.; Madgwick, T. D.; Markham, M. L.; Mollart, T. P. Chemical vapour deposition synthetic diamond: materials, technology and applications. *J. Phys. Condens. Matter* **2009**, *21*, 364221.

(S17) Chaudhuri, S.; Hall, S. J.; Klein, B. P.; Walker, M.; Logsdail, A. J.; Macpherson, J. V.; Maurer, R. J. Coexistence of carbonyl and ether groups on oxygen-terminated (110)-oriented diamond surfaces. *Commun. Mater.* **2022**, *3*, 6.

(S18) Newman, M. S. A notation for the study of certain stereochemical problems. *J. Chem. Educ.* **1955**, *32*, 344–347.

(S19) Ceperley, D. M.; Alder, B. J. Ground State of the Electron Gas by a Stochastic Method. *Phys. Rev. Lett.* **1980**, *45*, 566–569.

(S20) Perdew, J. P.; Zunger, A. Self-interaction correction to density-functional approximations for many-electron systems. *Phys. Rev. B* **1981**, *23*, 5048–5079.

(S21) Karasiev, V. V.; Sjostrom, T.; Dufty, J.; Trickey, S. B. Accurate Homogeneous Electron Gas Exchange-Correlation Free Energy for Local Spin-Density Calculations. *Phys. Rev. Lett.* **2014**, *112*, 076403.

(S22) Zhang, Y.; Yang, W. Comment on "Generalized Gradient Approximation Made Simple". *Phys. Rev. Lett.* **1998**, *80*, 890.

(S23) Hammer, B.; Hansen, L. B.; Nørskov, J. K. Improved adsorption energetics within density-functional theory using revised Perdew-Burke-Ernzerhof functionals. *Phys. Rev. B* **1999**, *59*, 7413–7421.

(S24) Perdew, J. P.; Ruzsinszky, A.; Csonka, G. I.; Vydrov, O. A.; Scuseria, G. E.; Constantin, L. A.; Zhou, X.; Burke, K. Restoring the Density-Gradient Expansion for Exchange in Solids and Surfaces. *Phys. Rev. Lett.* **2008**, *100*, 136406.

(S25) Tao, J.; Perdew, J. P.; Staroverov, V. N.; Scuseria, G. E. Climbing the Density Functional Ladder: Nonempirical Meta–Generalized Gradient Approximation Designed for Molecules and Solids. *Phys. Rev. Lett.* **2003**, *91*, 14601.

(S26) Constantin, L. A.; Fabiano, E.; Della Sala, F. Semilocal dynamical correlation with increased localization. *Phys. Rev. B* **2012**, *86*, 035130.

(S27) Perdew, J. P.; Ruzsinszky, A.; Csonka, G. I.; Constantin, L. A.; Sun, J. Workhorse Semilocal Density Functional for Condensed Matter Physics and Quantum Chemistry. *Phys. Rev. Lett.* **2009**, *103*, 026403.

(S28) Adamo, C.; Barone, V. Toward reliable density functional methods without adjustable parameters: The PBE0 model. *J. Chem. Phys.* **1999**, *110*, 6158–6170.

(S29) del Campo, J. M.; Gázquez, J. L.; Trickey, S. B.; Vela, A. Non-empirical improvement of PBE and its hybrid PBE0 for general description of molecular properties. *J. Chem. Phys.* **2012**, *136*, 104108.

(S30) Heyd, J.; Scuseria, G. E.; Ernzerhof, M. Hybrid functionals based on a screened Coulomb potential. *J. Chem. Phys.* **2003**, *118*, 8207–8215.

(S31) Krukau, A. V.; Vydrov, O. A.; Izmaylov, A. F.; Scuseria, G. E. Influence of the exchange screening parameter on the performance of screened hybrid functionals. *J. Chem. Phys.* **2006**, *125*, 224106.

(S32) Perdew, J. P.; Schmidt, K. Jacob's ladder of density functional approximations for the exchange-correlation energy. *AIP Conf. Proc.* **2001**, *577*, 1–20.
